# Comparative and phylogenetic analysis of *Chiloschista* (Orchidaceae) species and DNA barcoding investigation based on plastid genomes

**DOI:** 10.1186/s12864-023-09847-8

**Published:** 2023-12-06

**Authors:** Ding-Kun Liu, Cheng-Yuan Zhou, Xiong-De Tu, Zhuang Zhao, Jin-Liao Chen, Xu-Yong Gao, Shao-Wei Xu, Meng-Yao Zeng, Liang Ma, Sagheer Ahmad, Ming-He Li, Siren Lan, Zhong-Jian Liu

**Affiliations:** 1 College of Forestry, Fujian Colleges and Universities Engineering Research Institute of Conservation and Utilization of Natural Bioresources, Fujian Agriculture and Forestry University, Fuzhou, 350002 China; 2 Key Laboratory of Orchid Conservation and Utilization of National Forestry and Grassland Administration, College of Landscape Architecture and Art, Fujian Agriculture and Forestry University, Fuzhou, 350002 China; 3 Fujian Health College, Fuzhou, 350101 Fujian China

**Keywords:** Aeridinae, Chloroplast genome, DNA barcoding, Small inversions, Phylogenetic analysis

## Abstract

**Background:**

*Chiloschista* (Orchidaceae, Aeridinae) is an epiphytic leafless orchid that is mainly distributed in tropical or subtropical forest canopies. This rare and threatened orchid lacks molecular resources for phylogenetic and barcoding analysis. Therefore, we sequenced and assembled seven complete plastomes of *Chiloschista* to analyse the plastome characteristics and phylogenetic relationships and conduct a barcoding investigation.

**Results:**

We are the first to publish seven *Chiloschista* plastomes, which possessed the typical quadripartite structure and ranged from 143,233 bp to 145,463 bp in size. The plastomes all contained 120 genes, consisting of 74 protein-coding genes, 38 tRNA genes and eight rRNA genes. The *ndh* genes were pseudogenes or lost in the genus, and the genes *petG* and *psbF* were under positive selection. The seven *Chiloschista* plastomes displayed stable plastome structures with no large inversions or rearrangements. A total of 14 small inversions (SIs) were identified in the seven *Chiloschista* plastomes but were all similar within the genus. Six noncoding mutational hotspots (*trnN*^*GUU*^–*rpl32* > *rpoB*–*trnC*^*GCA*^ > *psbK*–*psbI* > *psaC*–*rps15* > *trnE*^*UUC*^*–trnT*^*GGU*^ > *accD*–*psaI*) and five coding sequences (*ycf1* > *rps15* > *matK* > *psbK* > *ccsA*) were selected as potential barcodes based on nucleotide diversity and species discrimination analysis, which suggested that the potential barcode *ycf1* was most suitable for species discrimination. A total of 47–56 SSRs and 11–14 long repeats (> 20 bp) were identified in *Chiloschista* plastomes, and they were mostly located in the large single copy intergenic region. Phylogenetic analysis indicated that *Chiloschista* was monophyletic. It was clustered with *Phalaenopsis* and formed the basic clade of the subtribe Aeridinae with a moderate support value. The results also showed that seven *Chiloschista* species were divided into three major clades with full support.

**Conclusion:**

This study was the first to analyse the plastome characteristics of the genus *Chiloschista* in Orchidaceae, and the results showed that *Chiloschista* plastomes have conserved plastome structures. Based on the plastome hotspots of nucleotide diversity, several genes and noncoding regions are suitable for phylogenetic and population studies. *Chiloschista* may provide an ideal system to investigate the dynamics of plastome evolution and DNA barcoding investigation for orchid studies.

**Supplementary Information:**

The online version contains supplementary material available at 10.1186/s12864-023-09847-8.

## Background

Species identification is crucial for biodiversity conservation, evolutionary analysis and molecular breeding [1–3]. DNA barcodes have been developed for species identification using DNA sequences from specific genes or intergenic regions [4, 5]. Barcoding facilitates quick and accurate identification of species with the benefits of uniformity, digitization, scalability and high reproducibility [6]. There are different DNA barcodes suitable for different taxa. The mitochondrial gene *cytochrome oxidase I* (*COI*) is the standard barcode for animals [7], but it is not suitable for plant species due to the low substitution rates [8]. Standardized barcodes have been used in plants. For example, the plastid genes *matK* and *rbcL* are used as the core barcodes for plant species identification, and the plastid intergenic sequences *trnL-F* and *trnH-psbA* serve as the spare barcodes [9]. Moreover, the internal transcribed spacer (ITS) of nuclear ribosomal DNA (nrDNA) comprises plant core barcodes, and it is also suitable for fungal species identification [10, 11]. The plant core barcodes are widely applied in most taxonomic studies [12, 13] but are limited in some taxonomically complex groups due to pseudogene amplification and radiation evolution [14–16].

An extended DNA barcode has been proposed using advanced genome sequencing technology [5, 17]. Genome skimming can concurrently obtain plastid genome (plastome), mitochondrial genome, and nrDNA sequences, which are congruent with standard DNA barcodes [17, 18]. The plastome is suitable and convenient for barcoding investigation and phylogenetic analysis due to its uniparental inheritance, moderate mutation rate and high copy number [19, 20]. The plastome also contains more informative sites than normal DNA barcodes and can be easily derived from genome skimming data by *de novo* assembly, which can work on herbarium specimens with degraded DNA [18, 21]. Therefore, all plastome sequences and nrDNA sequences are also known as ultrabarcodes or next generation barcodes [1, 22, 23]. Plastome barcodes may promote the classification and phylogenetic analysis of problematic taxonomic groups.

Orchidaceae is a taxonomically complex group that contains 28,000 species in 700 genera [24–26]. The standard barcodes (*matK*, *rbcL*, ITS) and supplementary barcodes *(psbA-trnH*, *trnL-F*, *atpI-H*) were applied in previous studies that resolved subfamily relationships and main clade relationships in some genera or subtribes [14–16, 27]. However, their use is limited in rapidly evolving orchids. For example, in the subtribe Aeridinae and related genera, five DNA barcodes have been used in phylogenetic analysis, providing a lower resolution tree due to radiation evolution [16, 28–30].

The genus *Chiloschista* Lindl. (1832), comprising approximately 20 species [31], is mainly distributed in China and the Indian subcontinent through Southeast Asia to Australia [32]. The species of *Chiloschista* are listed in the Convention on International Trade in Endangered Species (CITES). The orchids of *Chiloschista* are epiphytic with characteristic stemless and leafless bodies and are commonly known as shootless orchids [33]. The genus belongs to the subtribe Aeridinae of Orchidaceae, containing several genera (*Phalaenopsis* and *Vanda*) with high ornamental value. Previous phylogenetic studies indicated that *Chiloschista* was sister to *Dimorphorchis* and *Thrixspermum* in the subtribe Aeridinae basic clade A [32], but Zou et al. [16] recognized it as an independent secondary basic clade of Aeridinae. Therefore, phylogenetic analysis and barcoding investigation of *Chiloschista* may contribute to the classification of the Aeridinae subtribe.

The use of orchid plastomes has experienced explosive growth, especially in the past ten years, with the low-cost and widespread use of next-generation sequencing technology. A total of 474 plastomes have been released by NCBI [34], and the associated studies mainly concentrated on genome structure comparison, barcoding investigation, plastid phylogenomics and mycoheterotrophic plastome evolution [2, 3, 24, 35]. Recently, plastome studies have been performed on genome repeats, structural characteristics and mutation hotspots to investigate plastome barcodes, suggesting the effective use of plastome barcodes for species identification [3, 23, 36, 37]. Plastome data have been used to resolve the phylogenetic relationships of tribes, subtribes and genera in Orchidaceae [3, 24, 38–43], which further indicates that the plastomes are useful for phylogenetic relationship reconstruction. Moreover, plastomes have also been used to investigate the evolution of mycoheterotrophic orchid genomes suffering extreme gene loss and structural variation [43–47]. However, plastome analysis has yet to be implemented in leafless orchids, comprehensive plastome comparison and phylogenetic analysis in *Chiloschista*.

In this study, seven complete plastomes of *Chiloschista* were assembled, with the aim of investigating the plastome evolution, potential barcodes and phylogenetic relationships of the genus. We used plastome data to address the following specific questions: (1) What are the plastome characteristics of *Chiloschista* and the differences compared with other orchids? (2) What is the phylogenetic relationship (intergeneric and intrageneric) of *Chiloschista*, and how can useful barcodes be identified?

## Results

### Plastome characteristics and structure

The *k*-mer coverage of the seven *Chiloschista* species plastomes (accession number OP953683–OP953689) sequenced and assembled with Illumina reads was 108.5–122.8x (Table S1). Plastome sizes ranged from 143,223 bp for *C. pusilla* and 145,463 bp for *C. yunnanensis*, which all fell within the typical angiosperm plastome size range. Each plastome possessed the quadripartite structure common to angiosperm plastomes, with comparable percentages in each region (LSC 57.7–58.3%, IR 34.5–35.1%, and SSC 7.0–7.4%) and similar G/C contents (36.8–36.9%) (Table 1).

**Table 1:** Characteristics of the complete plastomes of the *Chiloschista* lineages

Species name	Size (bp)	GC content (%)	LSC size in bp (%)	IR size in bp (%)	SSC size in bp (%)	Total number of gene	Protein- coding gene	tRNA gene	rRNA gene	*ycf1* fragment (bp)	Number of *ndh* fragment
*Chiloschista exuperei*	144,909	36.9	84,191 (58.1)	25,297 (34.9)	10,124 (7.0)	120	74	38	8	69	5
* C. guangdongensis*	145,322	36.9	84,713 (58.3)	25,231 (34.7)	10,147 (7.0)	120	74	38	8	59	6
* C. lunifera*	145,004	36.8	83,684 (57.7)	25,390 (35.0)	10,540 (7.3)	120	74	38	8	148	4
* C. pusilla*	143,223	36.8	83,088 (58.0)	24,835 (34.7)	10,465 (7.3)	120	74	38	8	179	4
* C.* sp. 128	144,689	36.9	83,508 (57.7)	25,357 (35.1)	10,467 (7.2)	120	74	38	8	148	4
* C. viridiflava*	143,233	36.8	83,084 (58.0)	24,842 (34.7)	10,465 (7.3)	120	74	38	8	179	4
* C. yunnanensis*	145,463	36.9	84,497 (58.1)	25,107 (34.5)	10,752 (7.4)	120	74	38	8	0	6

**Fig. 1:**
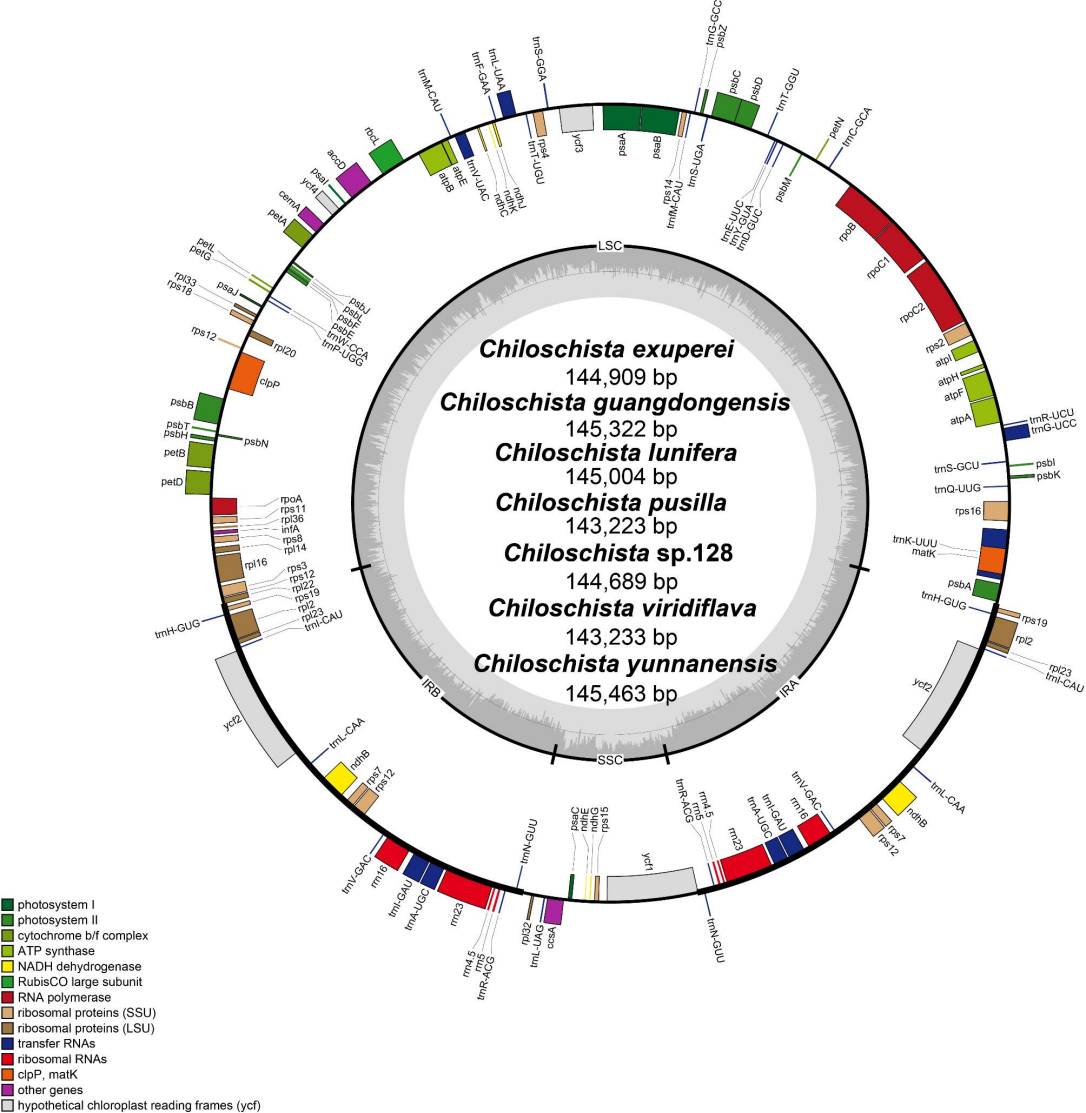
The annotation map of seven *Chiloschista* plastomes. The darker gray in the inner circle corresponds to the GC content. The IRA and IRB (two inverted repeating regions); LSC (large single-copy region); and SSC (Small single-copy region) are indicated outside of the GC content

A total of 120 genes (including repeat genes) were contained in each *Chiloschista* plastome, of which 74 were protein-coding genes, 38 were transfer RNA (tRNA) genes, and eight were ribosomal RNA (rRNA) genes (Table 1). Pseudogenization has occurred extensively in Aeridinae species (Li et al. 2019; Liu et al. 2020; Kim et al. 2020), wherein *ndh* genes were generally lost or truncated in *Chiloschista* (Fig. 1; Table 1). The *ndh* genes in the genus were all pseudogenes, ranging from four to six members in each plastome (Table 1). The plastomes of *C. guangdongensis*, *C. exuperei* and *C. yunnanensis* possessed six (*ndhB*/*C*/*E*/*G*/*J*/*K*), five (*ndhB*/*C*/*G*/*J*/*K*) and six (*ndhB*/*C*/*E*/*G*/*J*/*K*) pseudogenes, respectively. The other four species possessed four *ndh* pseudogenes (*ndhB*/*E*/*G*/*J*).

**Fig. 2:**
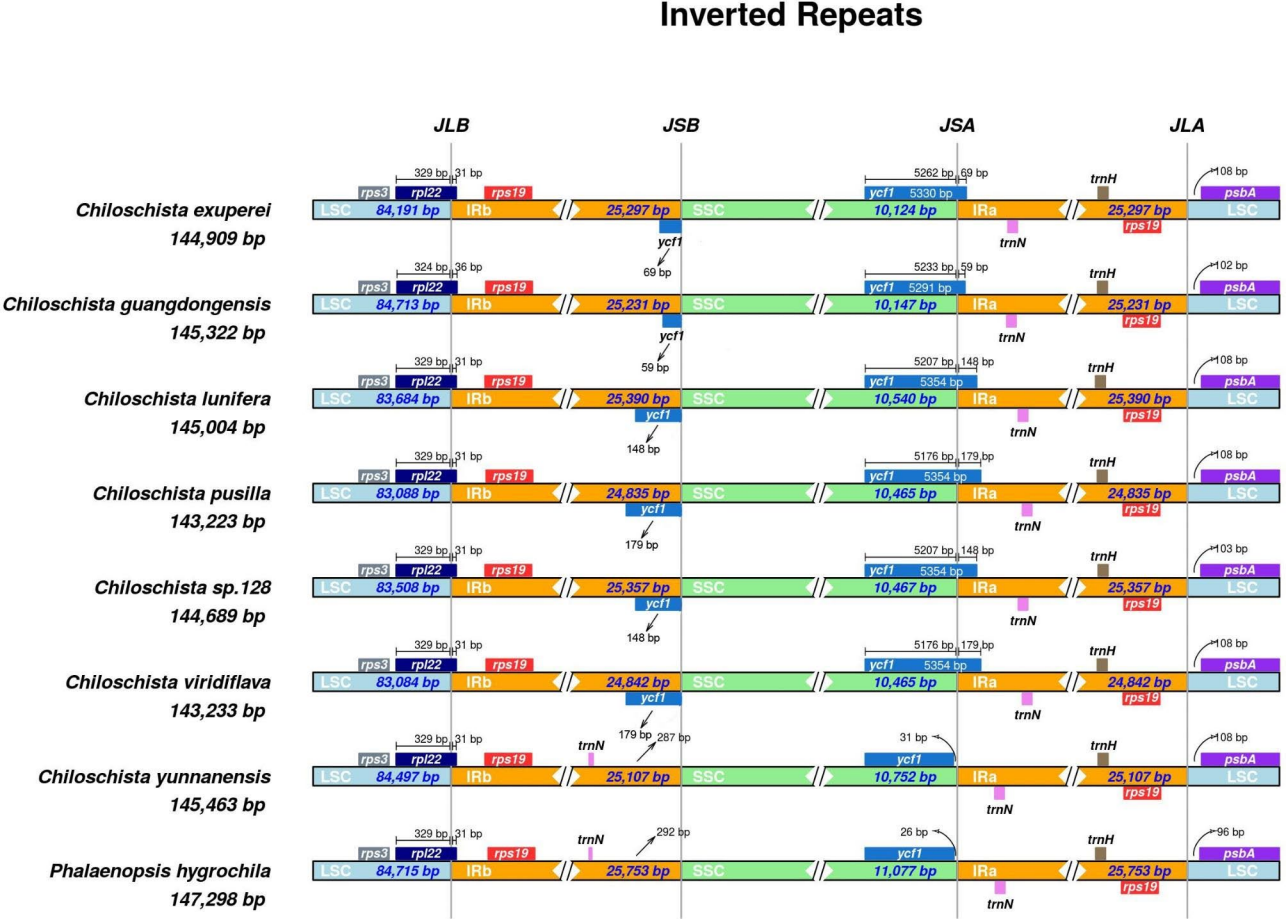
Comparison of junctions between the LSC, SSC, and IR regions among seven newly assembled *Chiloschista* plastomes and *Phalaenopsis hygrochila*

The results of IR boundary analysis indicated that JLA/B were more conserved than JSA/B (Fig. 2). The adjacent regions of the LSC and IRA (JLA) located at the *psbA* gene were similar in *Chiloschista*. The adjacent region of LSC and IRB (JLB) located at the *rpl22* gene was the same in *Phalaenopsis hygrochila* and *Chiloschista*. The adjacent region of SSC and IRA (JSA) containing the *ycf1 gene*, the genes of *C. yunnanensis* and *P. hygrochila*, was complete in the SSC region with no *ycf1* fragments in the adjacent region of SSC and IRB (JSB). The SSC regions of *C. yunnanensis* and *P. hygrochila* were expanded in the above two species.

**Fig. 3:**
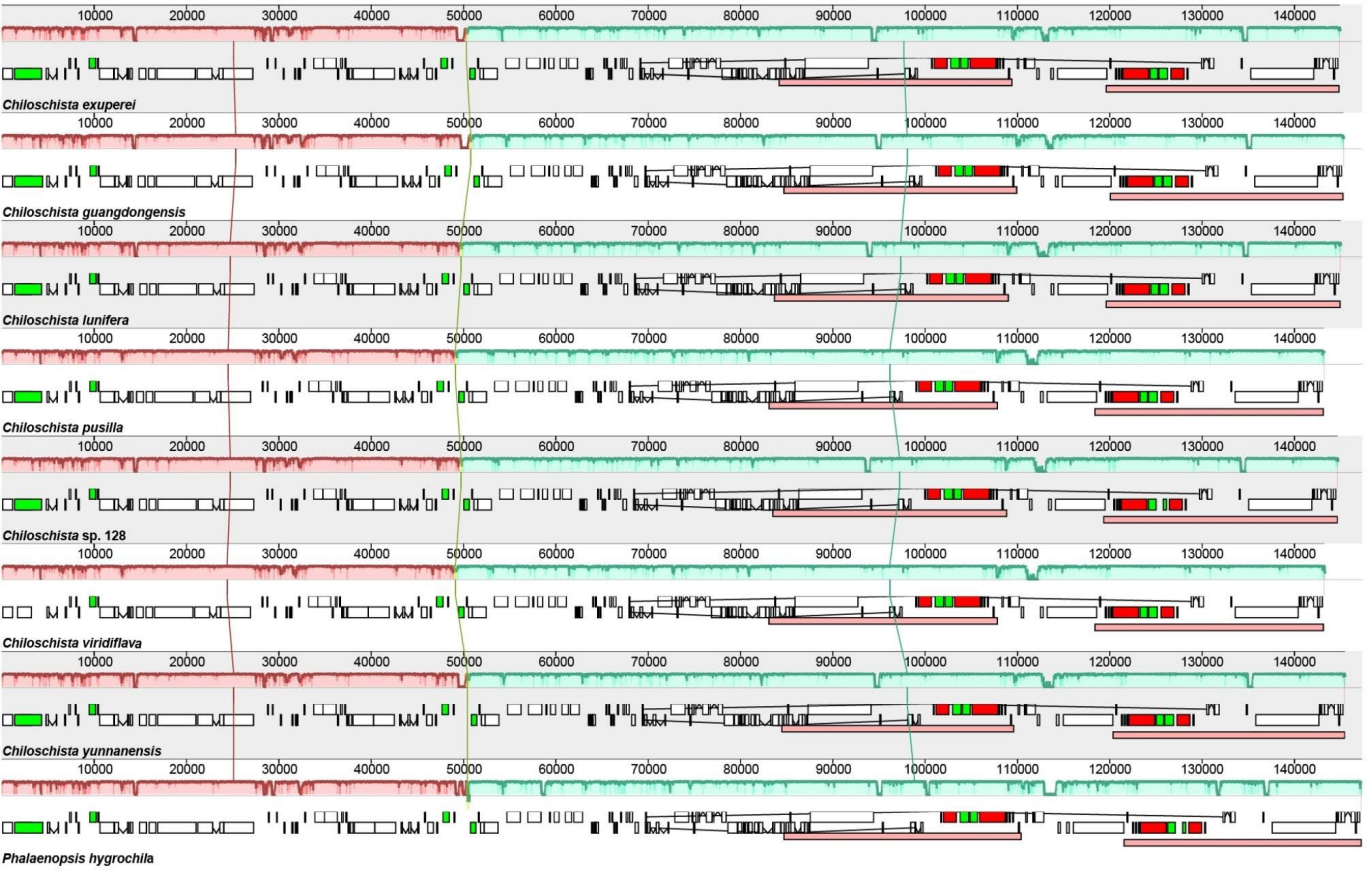
Plastome comparison of seven species of *Chiloschista* and *Phalaenopsis hygrochila* using a progressive MAUVE algorithm

**Fig. 4:**
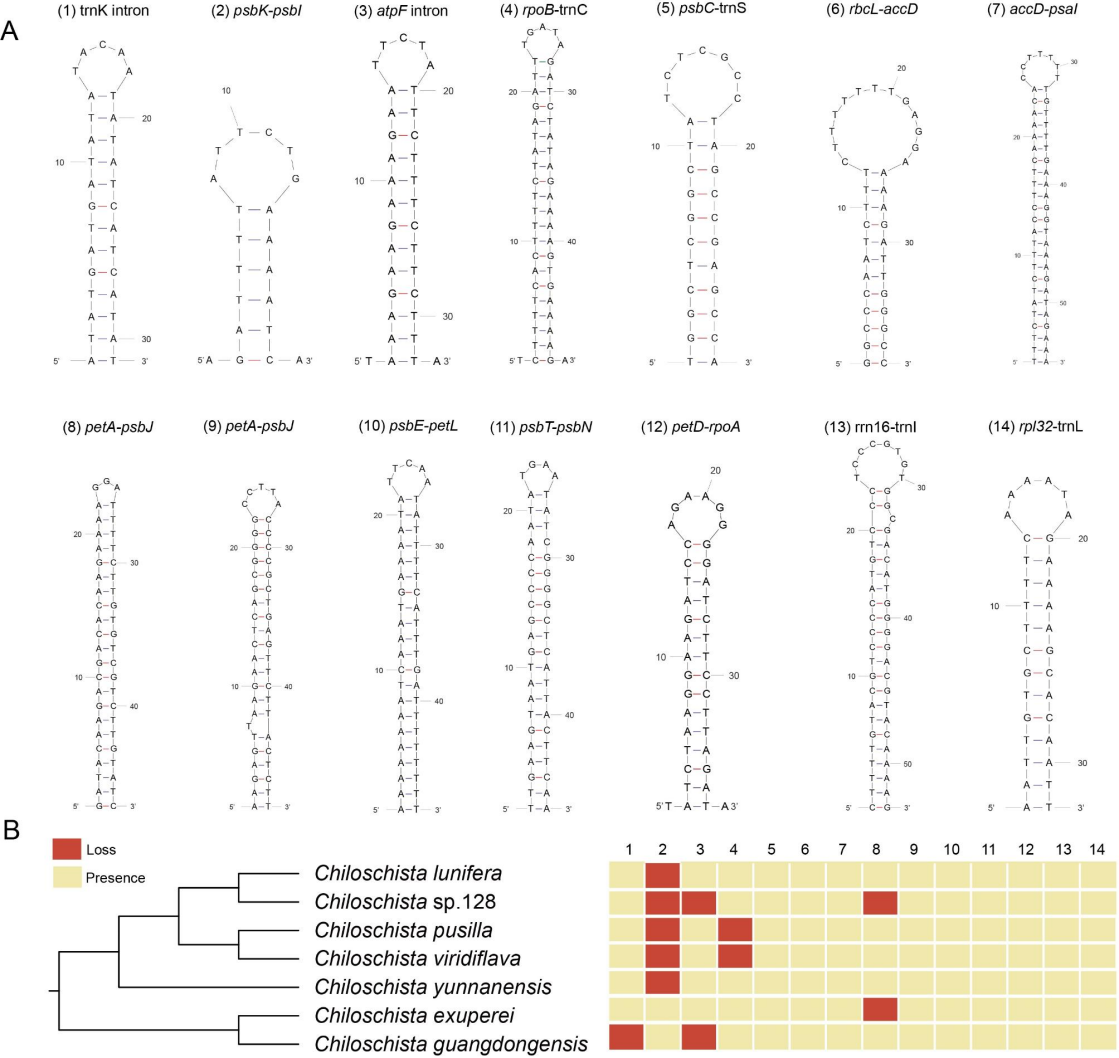
Stem-loop structure of fourteen small inversions across seven *Chiloschista* species. Major types are represented. Details of free energy, sequences, loop length, and subtypes are described in Table S3

The typical *Chiloschista* plastomes exhibited no inversions or rearrangements, but one inversion was identified compared with the *P. hygrochila* reference plastome (Fig. 3). Fourteen small inversions (SIs) were identified in the seven *Chiloschista* plastomes (Fig. 4; Table S2). The locations of all 14 SIs were in stem- and loop-forming regions, with 12 SIs being detected in the intergenic region and two in the intron (Fig. 4). The LSC region contained 12 SIs, and the IR and SSC regions contained one SI (Fig. 1). Those of the 14 primary SI types that have base substitutions in the stem or loop were catalogued as subtypes. The catalogue of subtypes in the stem or loop may easily identify the trait distribution states during plastome evolutionary processes. The dG value for each hairpin is displayed in Table S2, with high stability between different species. dG represents the quantity of energy needed to fully break a secondary DNA structure. The dG values also indicate the stability of each hairpin.

**Fig. 5:**
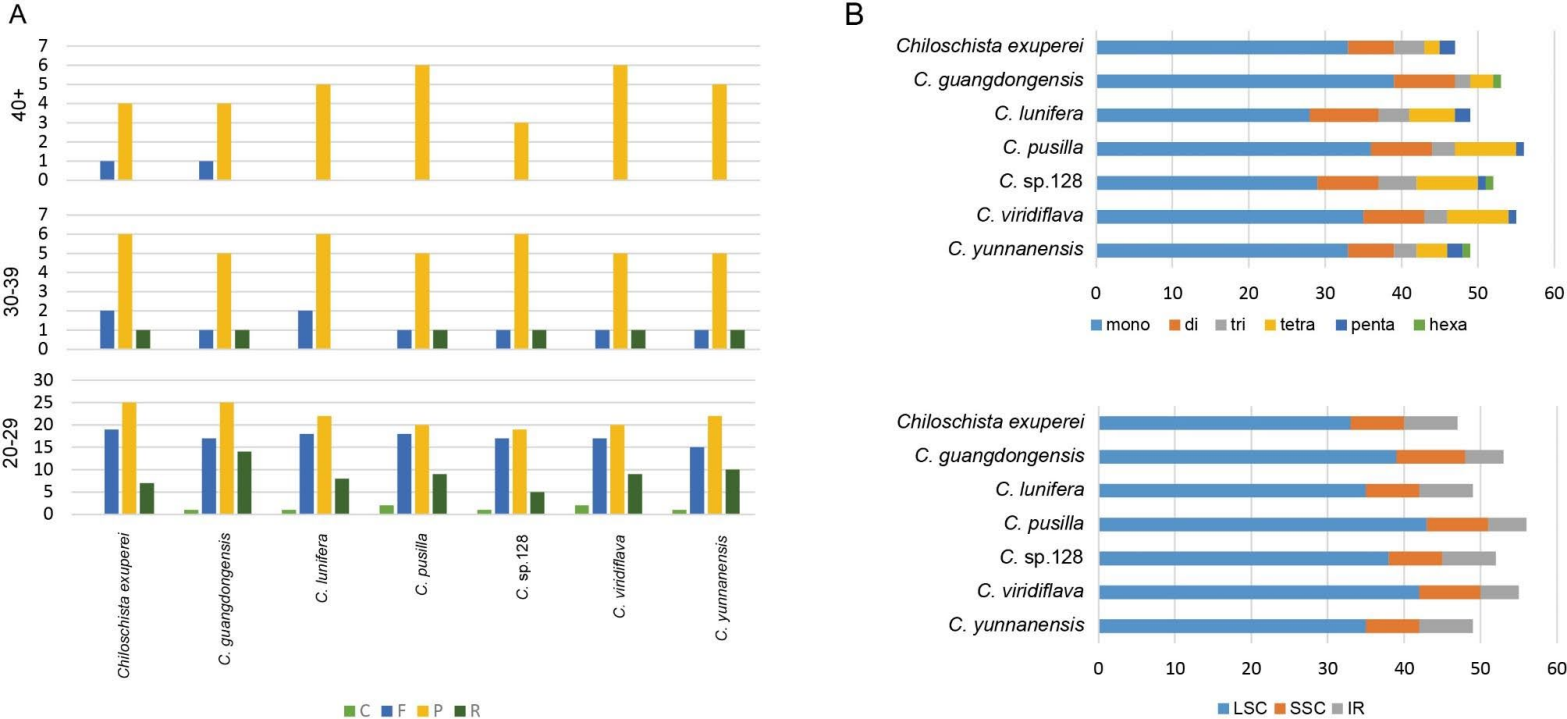
Summary of simple sequence repeats (SSR) across the *Chiloschista* species. (**A**) Variation in repeat abundance and type in seven plastomes. (**B**) Number of SSRs for each *Chiloschista* species by SSR unit size, and number of SSRs for each Aeridinae species by location in IR, LSC, and SSC

To clarify allied species or intragenus variations, the number of repeat sequences and regions of distribution were examined. First, four types of repeats (complement, forward, palindrome and reverse) were examined in *Chiloschista* plastomes (Fig. 5A, Table S3). The majority of the repeat sequences were in the 20–29 bp range, followed by 30–39 bp, then over 40 bp, with the fewest in the over 40 bp range. There were no C and R repeats found over 40 bp in length, and they were infrequent even in the smaller size ranges. In the 30–39 bp group, there were no C repeats, and R repeats were detected in most species (Fig. 5A). Additionally, we investigated regions in which six types (mono-, di-, tri-, tetra-, penta-, and hexa-) of SSRs were analysed. A total of 47 (*C. exuperei*) – 56 (*C. pusilla*) SSRs were found in *Chiloschista* (Fig. 5B, Table S4). The majority of SSRs were found in the LSC region, while few SSRs were found in the SSC region (Fig. 5B).

### Plastome sequence divergence, evolution and barcoding investigation

**Fig. 6:**
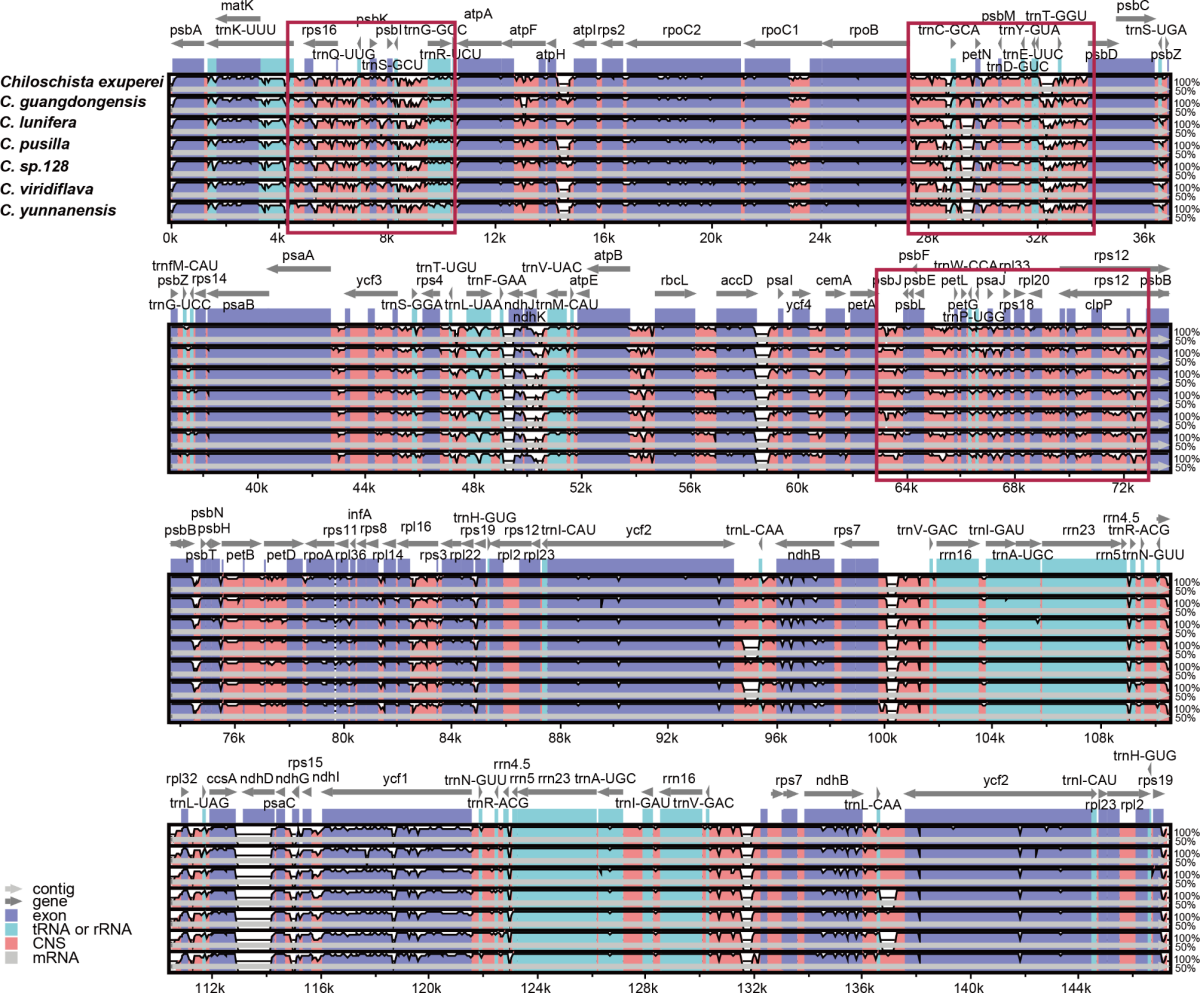
Global alignment of seven *Chiloschista* plastomes using mVISTA with *Phalaenopsis hygrochila* as reference. The y-axis shows the coordinates between the plastomes. The red boxes mean high variation regions in plastome sequence

We used mVISTA to find regions with high variations between conserved regions to further describe the differences between seven newly assembled *Chiloschista* species plastomes and that of *P. hygrochila* (outgroup species). High variation was identified in the intergenic and intragenic regions of plastomes in *Chiloschista* and *P. hygrochila* (Fig. 6), particularly in the LSC (from *rpoB* to *psbD*) and SSC regions (from *rpl32* to *ycf1*). As a result of these findings, multiple intergenic and intragenic regions may be suitable for DNA barcode investigations that can easily distinguish between *Chiloschista* species.

To investigate the evolutionary characteristics of the *Chiloschista* plastomes, we conducted a substitution rate comparison analysis of 68 protein-coding genes. Among the genes in *Chiloschista* plastomes, the estimated nonsynonymous (dN)/synonymous (dS) substitution rates were 0.00010–0.62310 under purifying selection, except for *petG* (2.13591) and *psbF* (3.87157) genes under positive selection (Fig. S1, Table S5).

**Fig. 7:**
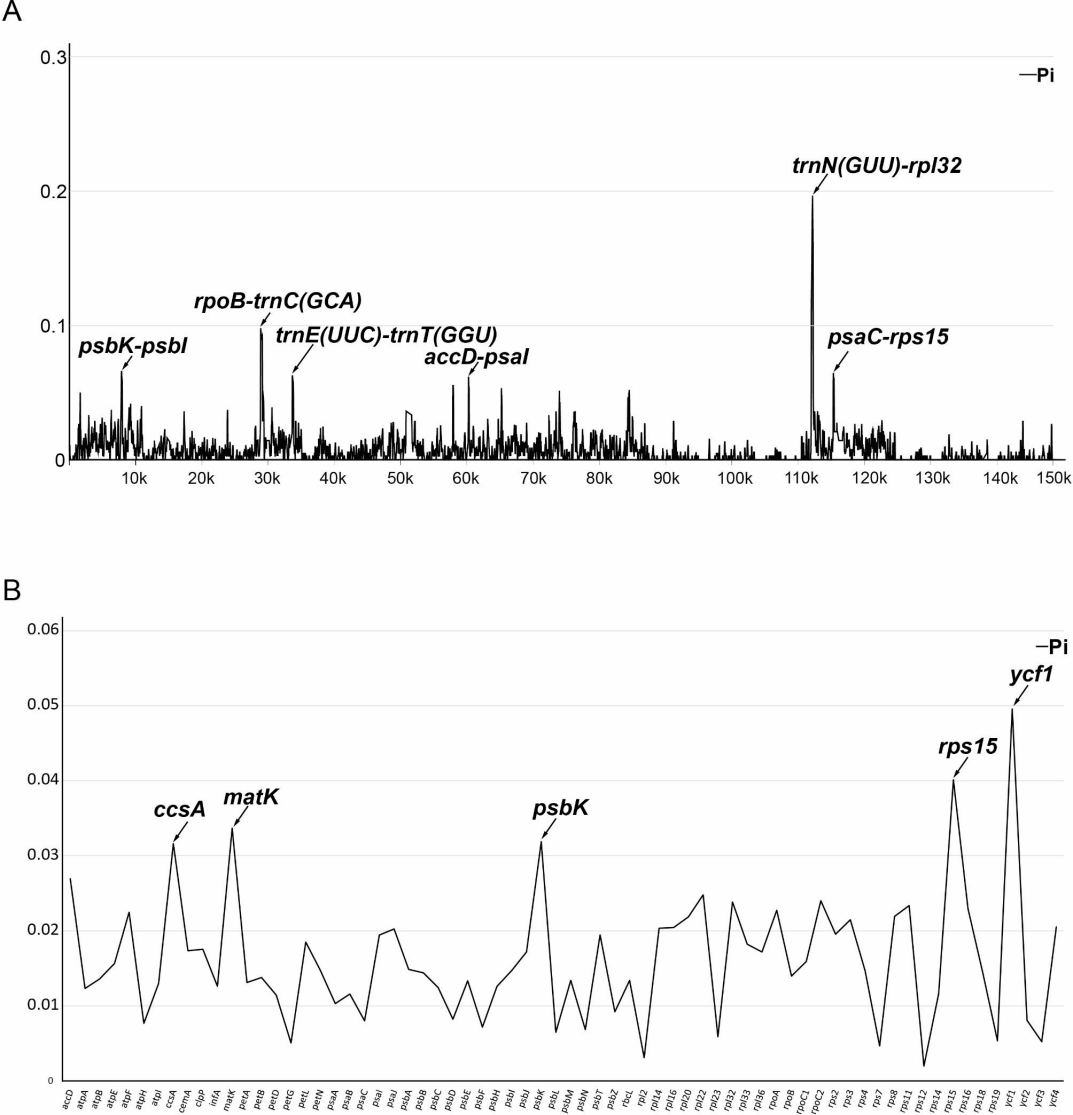
Sliding window test of nucleotide diversity (π) in the *Chiloschista* Plastomes. (**A**) The nucleotide diversity of complete plastome, six mutation hotspot regions (π > 0.06) were annotated. (**B**) The nucleotide diversity of 68 protein coding sequence, five mutation hotspot region (π > 0.03). The window size was set to 100 bp and the sliding windows size was 25 bp. X-axis, position of the midpoint of a window; Y-axis, π values of each window

To further analyse the mutation hotpots in the *Chiloschista* plastomes, we used DnaSP6 to assess the nucleotide diversity (Pi) of the alignment of the complete genome. The results showed high divergence of the SSC region and conservation of the IR region (Fig. 7, Tables S6, S7). We selected six mutational hotspots (Pi value: *trnN*^*GUU*^–*rpl32* (0.191) > *rpoB*–*trnC*^*GCA*^ (0.097) > *psbK*–*psbI* (0.065) > *psaC*–*rps15* (0.063) > *trnE*^*UUC*^–*trnT*^*GGU*^ (0.061) > *accD*–*psaI* (0.059)) for candidate barcodes. The protein-coding genes were also used for nucleotide diversity analysis. The results showed five coding sequences (Pi value: *ycf1* (0.050) > *rps15* (0.040) > *matK* (0.034) > *psbK* (0.032) > *ccsA* (0.031)) with high nucleotide diversity that were appropriate for phylogeny.

Eleven datasets were obtained to perform species discrimination analysis using the tree-building method (Table 2). The results showed that the lengths of the five coding sequence potential barcode (*ycf1*, *matK*, *ccsA*, *rps15*, *psbK*) matrices were 6,105, 1,595, 996, 276, and 192, respectively. The informative sites of the above five potential barcodes contained 784, 147, 90, 27, and 16 informative sites. The species discrimination rate (95.45%, 68.18%, 59.09%, 45.45%, 13.64%) corresponded with the number of informative sites in coding sequences. The results showed that the lengths of six noncoding sequence potential barcodes (*psbK–psbI*, *rpoB*–*trnC*^*GCA*^, *psaC*–*rps15*, *trnE*^*UUC*^–*trnT*^*GGU*^, *accD*–*psaI*, *trnN*^*GUU*^–*rpl32*) were 688, 2,048, 1,017, 1,846, 1,230, and 5,158, respectively. The informative sites of six noncoding potential barcodes contained 72, 278, 137, 259, 128, and 356 informative sites, which did not correspond to the species discrimination rates (76.19%, 71.43%, 68.75%, 66.67%, 40.91%, and 35.00%, respectively). The results showed that *ycf1* and *psbK-psbI*, with high species discrimination, were appropriate for use as barcodes.

**Table 2:** Evaluation of five coding genes and six noncoding sequences and ability to discriminate species

	*ycf1*	*matK*	*ccsA*	*rps15*	*psbK*	*psbK*–*psbI*	*rpoB–trnC* ^*GCA*^	*psaC*–*rps15*	*trnE*^*UUC*^–*trnT*^*GGU*^	*accD*–*psaI*	*trnN*^*GUU*^–*rpl32*
Length of aligned sequence (bp)	6105	1595	996	276	192	688	2048	1017	1846	1230	5158
No. of variable sites (bp)	1605	295	160	48	27	184	663	314	624	289	1034
No. of informative sites (bp)	784	147	90	27	16	72	278	137	259	128	356
No. of species samples	22	22	22	22	22	21	21	16	21	22	20
Species discriminate	95.45%	68.18%	59.09%	45.45%	13.64%	76.19%	71.43%	68.75%	66.67%	40.91%	35.00%

### Phylogenetic analysis

**Fig. 8:**
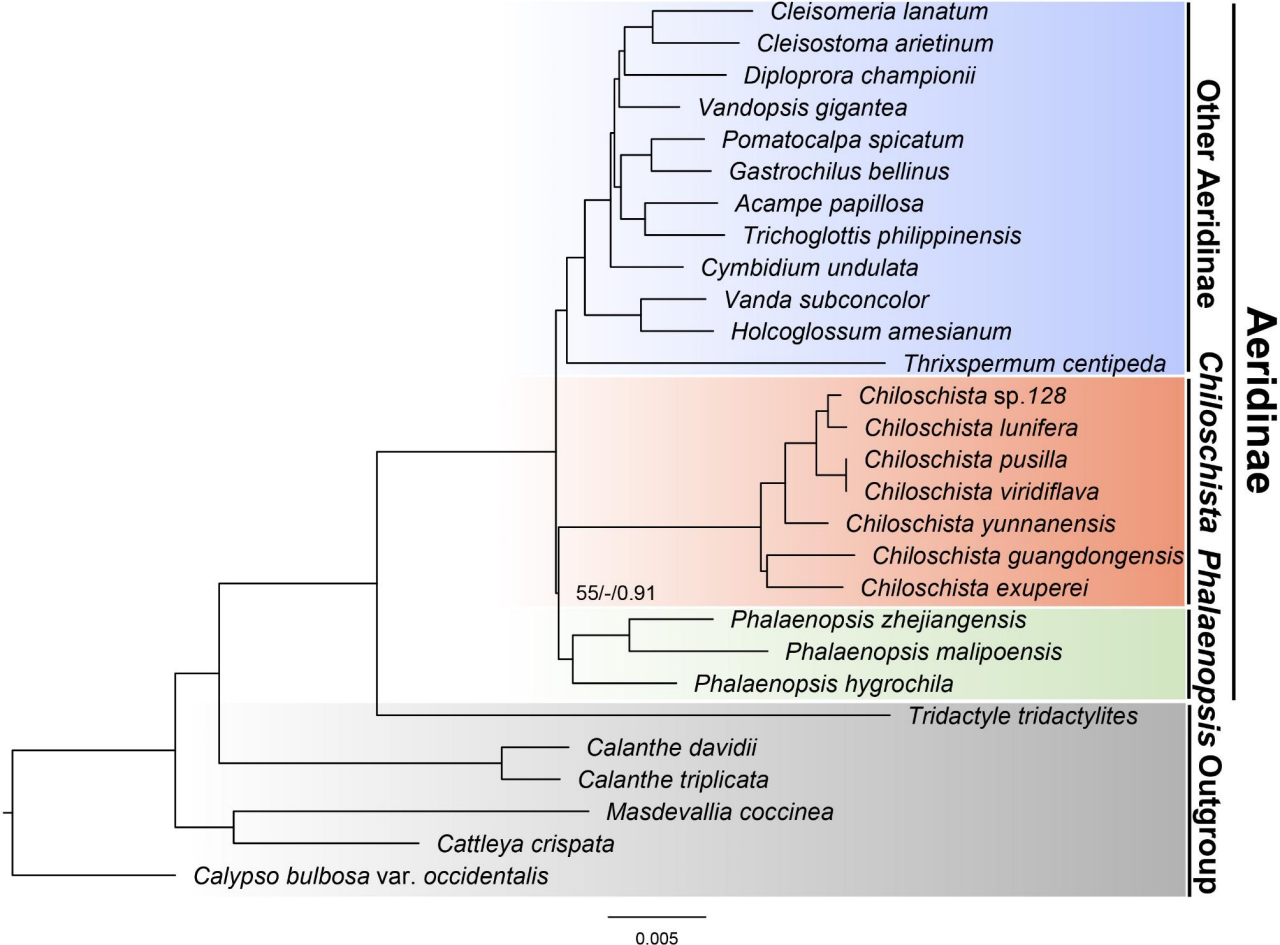
Phylogenetic tree of *Chiloschista* and other 15 Aeridinae species based on the complete plastome data. Numbers near the nodes are bootstrap percentages and Bayesian posterior probabilities (BS_ML_ left, BS_MP_ middle, and PP right). The nodes without values are 100% bootstrap or 1.00 posterior probability

The phylogenetic relationship analysis was conducted by three methods (ML, MP and BI) based on the whole plastome, and 68 protein-coding sequences resulted in a similar topology (Fig. 8; Fig. S2). Seven species of *Chiloschista* formed a monophyletic genus, which was clustered with *Phalaenopsis* at the basic clade of subtribe Aeridinae with moderate support values. The seven *Chiloschista* species were classified into three major clades. *C. guangdongensis* and *C. exuperei* formed the first clade of *Chiloschista*, *C. yunnanensis* was the second clade, and *C. viridiflava*, *C. pusilla*, *C.* sp. 128 and *C. lunifera* formed the third clade.

We also used informative noncoding sequences and coding sequences to reconstruct the phylogenetic relationship for useful barcode investigation. The informative noncoding sequence *trnN*^*GUU*^-*rpl32*, coding sequence *ycf1*, concatenation of six noncoding sequences, and concatenation of five coding sequences were used for phylogenetic analysis (Fig. S3). The phylogenetic analysis of *Chiloschista* based on *trnN*^*GUU*^-*rpl32* resulted in high support values but different relationships among *C. guangdongensis*, *C. exuperei* and *C. yunnanensis*. Three phylogenetic trees presented same topologies, and the tree based on five coding sequences had high support values.

## Discussion

### Plastome characteristics and structural evolution

Plastome sizes in Orchidaceae are diverse due to variable lifeforms, ranging from 19,047 bp (*Epipogium roseum*) to 212,688 bp (*Cypripedium tibeticum*) [48, 49]. The plastome size of *Chiloschista* was conserved, ranging from 143,233 bp to 145,643 bp, which is in accordance with the ranges of previously reported orchid plastomes. The GC content (36.8–36.9%) also fell within the range of 23.1% (*Gastrodia flexistyla*) to 37.8% (*Cypripedium macranthos*) [49, 50]. Plastome size in orchids is closely related to gene loss and IR boundary shifts [41, 51]. We found that the *ndh* genes of *Chiloschista* were pseudogenes (Fig. S4, Table 1), which is consistent with the plastome study of the subtribe Aeridinae [2, 38]. Previous studies suggested that the IR boundary shift was also related to plastome size; the *Pelargonium* plastomes ranged from 165,508 bp to 242,575 bp, and IR expansion or contraction was the main contribution [52–54]. The present study revealed that the IR boundary JLA/B was conserved compared with JSA/B (Fig. 2), while compared with other orchids, the *Chiloschista* IR boundary was conserved [41, 49]. The plastome size of *Chiloschista* was relatively conserved, and the variation could be contributed by *ndh* loss instead of the IR boundary shift.

Plastome protein-coding gene loss is usually found in orchids, and mycoheterotrophic orchids obtain energy from fungi, leading to plastome degradation [44, 45, 48, 55]. However, autotrophic orchids also suffer gene loss on some occasions [2, 38, 56, 57]. We annotated 120 genes (containing repeat genes) in *Chiloschista*, and the gene number was less than that of other autotrophic orchids due to *ndh* gene loss. The *ndh* gene loss was general in epiphytic orchids, and previous studies suggested that the *ndh* loss did not correlate to taxonomic or evolutionary relationships [36, 56–58]. We also analysed *ndh* gene loss/pseudogenization in the evolutionary process. The results of Aeridinae did not exhibit an obvious *ndh* deletion pattern, as in previous studies [2, 38]. However, we found that *ndh* loss in *Chiloschista* can be divided into two parts: *ndhC/K* was absent in four species in a clade, and three species in basic clades were pseudogenes (Fig. S4). Our results suggested that *ndh* gene loss or pseudogenization might be accompanied by *Chiloschista* evolution. We also conducted substitution rate analysis to compare the evolution of genes. The results showed that the *petG* and *psbF* genes were under positive selection, and the other genes were under purifying selection. A previous study suggested that genes under positive selection may affect photosynthetic efficiency [59], and whether they can benefit *Chiloschista* adaptation needs more study in the future.

Plastome inversions are widespread in flowering plants [60–62], and recent studies have also found that they are universal in orchids [2, 41, 43]. Distinct inversions were not detected in *Chiloschista* in this study, but compared with the reference plastome of *Phalaenopsis hygrochila*, there was an inversion of approximately 500 bp in the intergenic region of *rps4*–*atpE* (Fig. 3); this inversion could be unique to the genus. We also performed small inversion/hairpin inversion (SI) analysis. SIs are ubiquitous in the angiosperm plastome and are always flanked by inverted repeats of approximately 8 to 50 bp [63]. There were 14 SIs detected in *Chiloschista* plastomes (Fig. 4, Table S2), which was consistent with the study of Aeridinae [38]. In each species of *Chiloschista*, there ranged from 11 to 13 SIs, and no phylogenetic signal was observed (Fig. 4). Eight SIs (3, 6, 7, 9, 10, 11, 12, 13) were different between *Chiloschista* plastomes (Table S2). A previous study indicated that intergeneric SI was easily distinguished [38], and based on our results, SIs may not be easily distinguished intrageneric relationships.

### Phylogenetic analysis and barcoding investigation

Our results revealed the phylogenetic position of *Chiloschista* and the intrageneric relationships. Few studies have been conducted on the molecular phylogenetics of *Chiloschista.* Carlsward et al. [33] indicated that *Chiloschista* was sister to the clade of four species (*Amesiella philippinensis*, *Tuberolabium kotoense*, *Dyakia hendersoniana* and *Tuberolabium brevirachis*), and the intrageneric relationship was ((*C. lunifera*, *C. pusilla*) *C. viridiflava*) *C. parishii*). Topik et al. [64] suggested that *C. viridiflava* was sister to *Ornithochilus difformis* (accepted name *Phalaenopsis difformis*) and belonged to the *Pelatantheria* alliance based on ITS and *matK*. Pridgeon et al. [32] suggested that *Chiloschista* was embedded in *Dimorphorchis* and sister to *Thrixspermum* formed the basic clade of Aeridinae. According to Zou et al. [16], *Chiloschista* was an independent clade of Aeridinae, and the intrageneric relationship was (*C.* sp. 4516 (*C. yunnanensis* (*C. lunifera* (*C. pusilla*, *C. parishii*)))). Previous studies documented that the relationships of *Chiloschista* usually exhibited unstable topology and low support values. Our results suggested that *Chiloschista* was an independent clade of Aeridinae, and it was clustered with *Phalaenopsis* at the basic clade with moderate support values (55/-/0.91) (Fig. 8). The relationship was not consistent with previous studies [16, 32], and the moderate support value implied that the *Chiloschista* could have different topology. We showed the full support values of the intrageneric relationship of *Chiloschista*, which provided new insight into relationship clarification.

Moreover, we performed nucleotide diversity analysis of the complete plastome and coding genes to investigate useful DNA barcodes for phylogenetic analysis. Plastomic mutational hotpots are convenient and practical regions for DNA barcoding development, as suggested by previous studies in orchids [3, 23, 37, 65–67]. The complete plastome nucleotide diversity analysis suggested that the SSC region was more variable than the LSC and IR regions (Figs. 6 and 7). Six intergenic regions (*trnN*^*GUU*^–*rpl32*, *rpoB*–*trnC*^*GCA*^, *psbK*–*psbI*, *psaC*–*rps15*, *trnE*^*UUC*^–*trnT*^*GGU*^, *accD*–*psaI*, Pi > 0.06) were selected for phylogenetic analysis (Fig. 2, Table S6). The results suggested that the potential barcodes possessed high diversity but might not be suitable for phylogenetic analysis due to the unstable topology. Based on the nucleotide diversity of protein-coding genes, five coding genes (*ycf1*, *rps15*, *matK*, *psbK*, *ccsA*, Pi > 0.03) were selected for phylogenetic analysis (Fig. S2, Table S7). The results based on five genes showed relatively high diversity and suitability for phylogenetic analysis. We also evaluated the potential barcodes of five coding sequences and six noncoding sequences for species discrimination (Table 2). The results indicated that the potential candidate barcode *ycf1* may be suitable for Aeridinae species discrimination. The applicability of this potential barcode to this group will be further evaluated through the identification of similar species in subsequent studies.

High mutational regions also contain SSRs (simple sequence repeats) and large repeats, which are widely used in studies of genetic diversity, population structure and species identification [68–70]. A total of 47–56 SSRs and 11–14 long repeats (> 30 bp) were identified in *Chiloschista* plastomes, which were mostly located in the intergenic region of LSC (Fig. 5, Tables S3, S4). The repeats in the coding regions were mainly located in the exons of *accD*, *rpoC2*, *ycf1* and *ycf2*. Most of the SSR types were mononucleotide repeats in the seven *Chiloschista* species. The mVISTA percent identity plot and sliding window analysis showed that the most divergent regions were located in regions of the *Chiloschista* plastomes. The results provide a data basis for future population genetics studies.

## Conclusions

In this study, we obtained the complete plastomes of seven *Chiloschista* species (*C. exuperei*, *C. guangdongensis*, *C. lunifera*, *C. pusilla*, *C.* sp. 128, *C. viridiflava* and *C. yunnanensis*) and found that the plastomes of the seven species possessed a generally preserved overall structure and gene content. Genome sizes, GC contents, gene contents, repeats and IR boundary variations showed little variance. It is important to note that all *ndh* genes in the plastomes of *Chiloschista* were deleted or truncated, as was also found in other species of the subtribe Aeridinae. The genes *petG* and *psbF* were under positive selection. We offer a resource for creating DNA barcodes to advance research on *Chiloschista* species, which suggests that the potential barcode *ycf1* is most suitable for species discrimination. Based on the available data, phylogenetic analysis was performed to identify the genus *Chiloschista* in the subtribe Aeridinae and, to a considerable extent, to establish the phylogenetic relationships of the majority taxonomic groups in the subtribe and above subtribe of Orchidaceae. These discoveries help us better comprehend the characteristics and evolution of *Chiloschista* plastomes, which further our knowledge of phylogenetic relationships and DNA barcoding for Aeridinae species conservation and even extend to the Orchidaceae family.

## Methods

### Taxon sampling and sequencing

Plant materials of seven *Chiloschista* species were collected from the Forest Orchid Garden greenhouse at Fujian Agriculture and Forestry University (Fuzhou, Fujian Province, China). The formal identification of plant material was conducted by Dr. Ming-He Li and Prof. Zhong-Jian Liu. The voucher specimen of materials was deposited at the Herbarium of Fujian Agriculture and Forestry University, and deposition numbers and GenBank accessions of species are listed in Table S8. A total of 28 species in 19 genera were analysed in combination with publicly accessible plastome data, including six species from five genera (*Calanthe*, *Calypso*, *Cattleya*, *Masdevallia* and *Tridactyle*) serving as the outgroup. According to the manufacturer’s protocol, total DNA was extracted from fresh leaves using the Plant Mini Kit (Qiagen. CA. USA), and DNA degradation and contamination were examined by 1% agarose gel electrophoresis. According to the manufacturer’s instructions, the HiSeq 4000 PE Cluster Kit (Illumina) was used to cluster the index-coded sample data on a cBot Cluster Generation System. The library preparations were sequenced on an Illumina HiSeq 4000 platform following cluster creation, and 150-bp paired-end reads were produced [71]. Scripts were used to filter the Illumina data in the cluster (default parameter: -L 5, -p 0.5, -N 0.1). When the low-quality (Q < = 5) base number in sequencing reads surpassed 50% of the read base number and the N content in reads exceeded 10% of the read base number, paired reads were eliminated from the analysis.

### Plastome assembly and annotation

To obtain plastid-like reads, the paired-end reads were filtered using the GetOrganelle pipeline (https://github.com/Kinggerm/GetOrganelle) [72]. The filtered reads were assembled using SPAdes version 3.10 [73]. To acquire pure plastid contigs, the final “fastg” files were filtered by the GetOrganelle script. The filtered De Bruijn graphs were then examined and corrected by Bandage [74]. The circular plastome was obtained through the above steps.

GeSeq [75] was used to annotate the newly assembled plastomes, while tRNAscan-SE v2.0.3 [76] was used to further verify tRNA genes. The start and stop codons in protein-coding genes found by GeSeq were manually visualized and corrected by alignment with the plastomes of related species in Geneious R11.1.5 [77]. The translation of each protein-coding gene was also validated by Geneious R11.1.5 [77]. A gene containing one or several internal stop codons compared to homologous genes was determined to be a pseudogene or partial copy. The plastome annotation file was generated using GB2Sequin [78], which was submitted to GenBank at the National Center for Biotechnology Information (NCBI) with unique accession numbers. The circle diagrams of annotated plastomes were drawn by OGDRAW [79]. The genes with ≥ 50% loss of the complete CDSs or similarity ≤ 50% were considered lost genes [38].

### Plastome structure analysis

REPuter [80] was used to identify the long repeats of seven *Chiloschista* plastomes with default parameters, and four repeat types (F, forward, P, palindrome, R, reverse, and C, complement) were identified. The Perl script of MISA [81] was used to identify simple sequence repeats (SSRs) with minimal thresholds of 10, 5, 4, 3, 3, and 3 repeat units for mono-, di-, tri-, tetra-, penta-, and hexa-motif microsatellites, respectively. The collinearity and rearrangements of plastomes were analysed and drawn by Mauve [82]. The boundary expansion or contraction analysis between the inverted repeat (IR) and single-copy (SC) regions was compared using Geneious 11.1.5 and IRscope [76, 83].

### Sequence divergence and barcoding investigation

The online tool mVISTA [84] was used to analyse the plastome sequence diversity through the comparison of seven *Chiloschista* plastomes by the Shuffle-LAGAN alignment program [85]. The *P. hygrochila* plastome was used as a reference. Complete chloroplast genomes and 68 coding sequences of seven *Chiloschista* species alignment files were used to analyse the nucleotide diversities (Pi) with a window length of 100 sites and a step size of 25 sites by DnaSP 6 [86]. According to the Pi value, five protein-coding genes and six noncoding sequences were selected for species discrimination analysis. The tree-building method was adopted to analyse the eleven datasets. For the tree-building method, all datasets were aligned by MAFFT software [87], and the maximum likelihood (ML) tree was constructed by IQTREE [88, 89].

### Phylogenetic analysis

The phylogenetic analysis of *Chiloschista* and other Aeridinae species was conducted based on the whole plastome and 68 protein-coding sequences. The whole plastome sequences were aligned by MAFFT [87]. The protein-coding sequences (*ndh* genes were widely lost or truncated in Aeridinae species) were aligned using MEGA 7.0 [90]. The alignment of the whole plastome was trimmed using trimAl v1.2 [91] with a heuristic approach (-automated1) to choose the best-automated method to decrease the systematic errors produced from poor quality. The online tool CIPRES Science Gateway (RaxML-HPC2 on XSEDE 8.2.12, PAUP on XSEDE 4.a168 and MrBayes on XSEDE 3.2.7) was used to perform phylogenetic analysis with three methods, including maximum likelihood (ML), maximum parsimony (MP) and Bayesian inference (BI) [92]. For MP analysis, 1000 tree-bisection-reconnection (TBR) searches with MAXTR-EES set to increase without limit were performed on the combined dataset by PAUP [93]. A heuristic search using 1000 random addition sequence repeats and TBR branch switching was conducted with all characters being equally weighted and unordered. For ML analysis, 1000 repeated self-expanding analyses using the GTRCAT model were carried out on all datasets [94]. For Bayesian analysis, the GTR + I + Γ substitution model was performed by MrBayes v. 3.2.7 [95]. The Markov chain Monte Carlo (MCMC) algorithm was run for 10,000,000 generations, with one tree sampled every 100 generations. To construct majority-rule consensus trees and estimate posterior probability (PP), the first 25% of trees were eliminated as burn-in.

### Nucleotide substitution rate analysis

The sequences of 68 protein-coding genes were retrieved from the *Chiloschista* plastomes to investigate the nucleotide substitution rate (Table S5). The branch model was selected for nucleotide substitution rate analysis. The *Chiloschista* clade was the foreground clade, and the others were background clades. The values of dN/dS (LRT: M0 vs. M2) in plastid protein-coding genes were estimated by EasyCodeML v1.0 [96].

### Electronic supplementary material

Below is the link to the electronic supplementary material.

**Supplementary Material 1: Fig. S1.** Comparison of non-synonymous (dN) / synonymous (dS) substitution rates among *Chiloschista* plastid protein-coding genes. **Fig. S2.** Phylogenetic analysis of 22 Aeridinae species based on 68 protein coding genes. **Fig. S3.** Phylogenetic analysis of 22 Aeridinae species based on six noncoding barcodes and five coding barcodes. **Fig. S4.** The *ndh* genes loss across the subtribe Aeridinae. **Table S1.** The statistics of raw data and plastome assembly results. **Table S2.** The description small inversion sequences of seven *Chiloschista*. **Table S3.** The details information of long repeats. **Table S8.** A list of the taxa analysed with voucher information and GenBank accessions

**Supplementary Material 2: Table S4.** The details information Small simple repeats. **Table S5.** Comparison of non-synonymous (dN) / synonymous (dS) substitution rates among *Chiloschista* plastid protein-coding genes. **Table S6.** The nucleotide diversity of seven *Chiloschista* plastome. **Table S7.** The nucleotide diversity of 68 protein coding genes in *Chiloschista*

## Data Availability

The plastome sequences of *Chiloschista* are openly available in NCBI at the GenBank database with accession number OP953683–OP953689, the raw data has been submitted to the SRA database (BioSample: SAMN37279563–SAMN37279569; BioProject: PRJNA1012891; SRA: SRR25905744–SRR25905750).

## Abbreviations

BI: Bayesian inference
IR: Invert repeat
ITS: Internal transcribed spacer
LSC: Large single copy
ML: Maximum likelihood
MP: Maximum parsimony
nrDNA: Nuclear ribosomal DNA
SI: Small inversion
SSC: Small single copy
SSR: Simple sequence repeats
