# Supplementary material for: Comparative and phylogenetic analysis of Chiloschista (Orchidaceae) species and DNA barcoding investigation based on plastid genomes
Source: BMC Genomics. 2023 Dec 6;24:749. doi: 10.1186/s12864-023-09847-8 (PMC10702055; doi:10.1186/s12864-023-09847-8)
Supplement: Supplementary file 1 — Supplementary Material 1: Fig. S1. Comparison of non-synonymous (dN) / synonymous (dS) substitution rates among Chiloschista plastid protein-coding genes. Fig. S2. Phylogenetic analysis of 22 Aeridinae species based on 68 protein coding genes. Fig. S3. Phylogenetic analysis of 22 Aeridinae species based on six noncoding barcodes and five coding barcodes. Fig. S4. The ndh genes loss across the subtribe Aeridinae. Table S1. The statistics of raw data and plastome assembly results. Table S2. The description small inversion sequences of seven Chiloschista. Table S3. The details information of long repeats. Table S8. A list of the taxa analysed with voucher information and GenBank accessions [file 12864_2023_9847_MOESM1_ESM.docx]

**Supplementary Figures and Tables**

**Fig. S1.** Comparison of non-synonymous (dN) / synonymous (dS) substitution rates among *Chiloschista* plastid protein-coding genes.

**Fig. S2.** Phylogenetic analysis of 22 Aeridinae species based on 68 protein coding genes.

**Fig. S3.** Phylogenetic analysis of 22 Aeridinae species based on six noncoding barcodes and five coding barcodes.

**Fig. S4.** The *ndh* genes loss across the subtribe Aeridinae.

**Table S1.** The statistics of raw data and plastome assembly results.

**Table S2.** The description small inversion sequences of seven *Chiloschista*.

**Table S3.** The details information of long repeats.

**Table S4.** The details information Small simple repeats.

**Table S5.** Comparison of non-synonymous (dN) / synonymous (dS) substitution rates among *Chiloschista* plastid protein-coding genes.

**Table S6.** The nucleotide diversity of seven *Chiloschista* plastome.

**Table S7.** The nucleotide diversity of 68 protein coding genes in *Chiloschista*.

**Table S8.** A list of the taxa analysed with voucher information and GenBank accessions.

**Supplementary Figures**


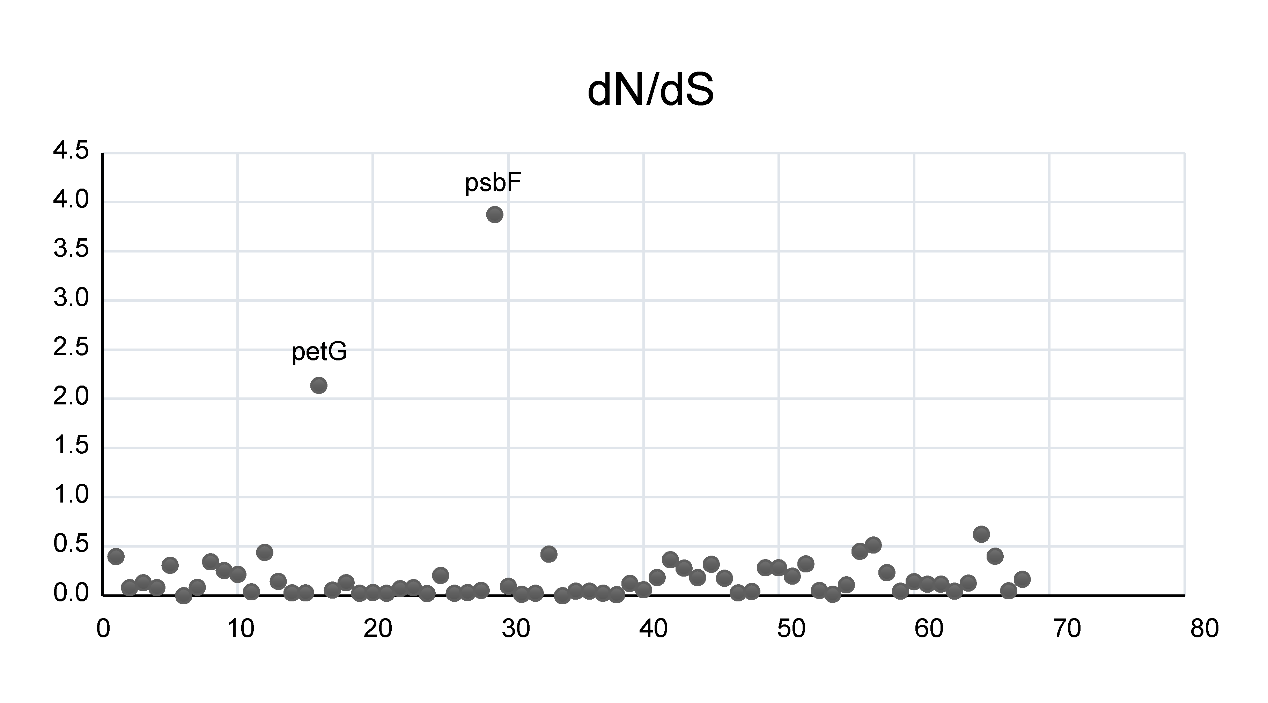


**Fig. S1.** Comparison of non-synonymous (dN) / synonymous (dS) substitution rates among *Chiloschista* plastid protein-coding genes.

**
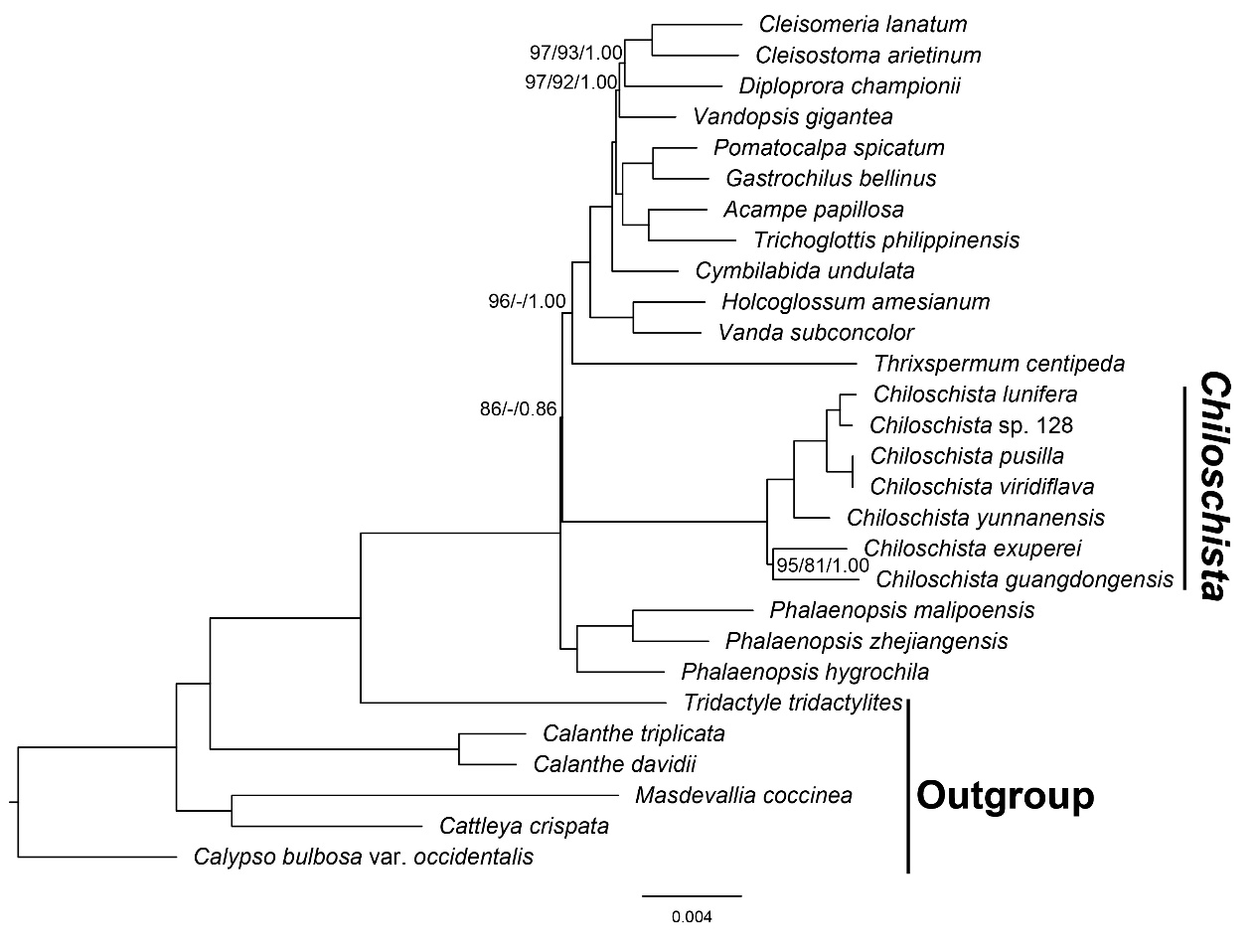
**

**Fig. S2.** Phylogenetic analysis of 22 Aeridinae species based on 68 protein coding genes.

**
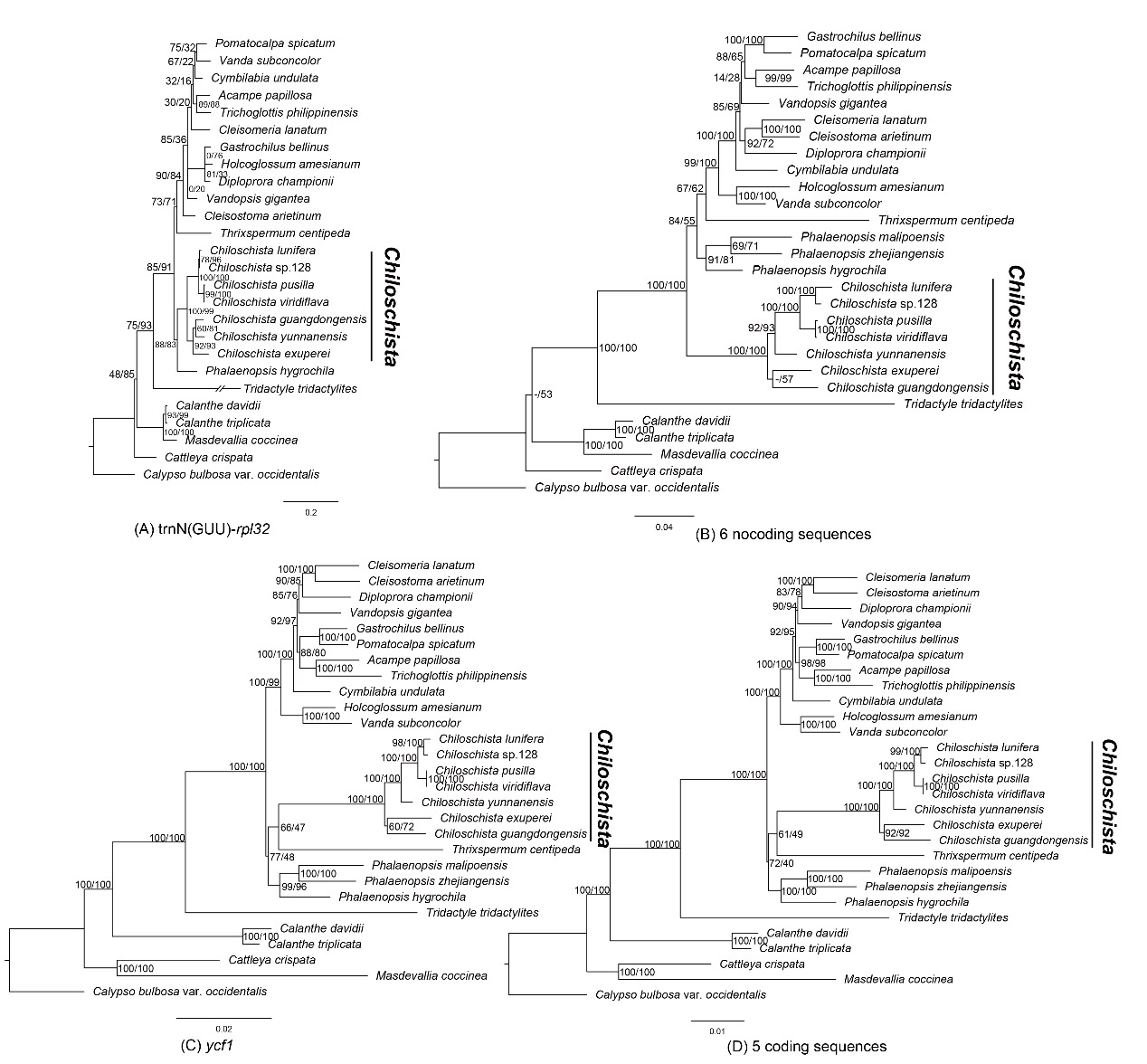
**

**Fig. S3.** Phylogenetic analysis of 22 Aeridinae species based on six noncoding barcodes and five coding barcodes.

**
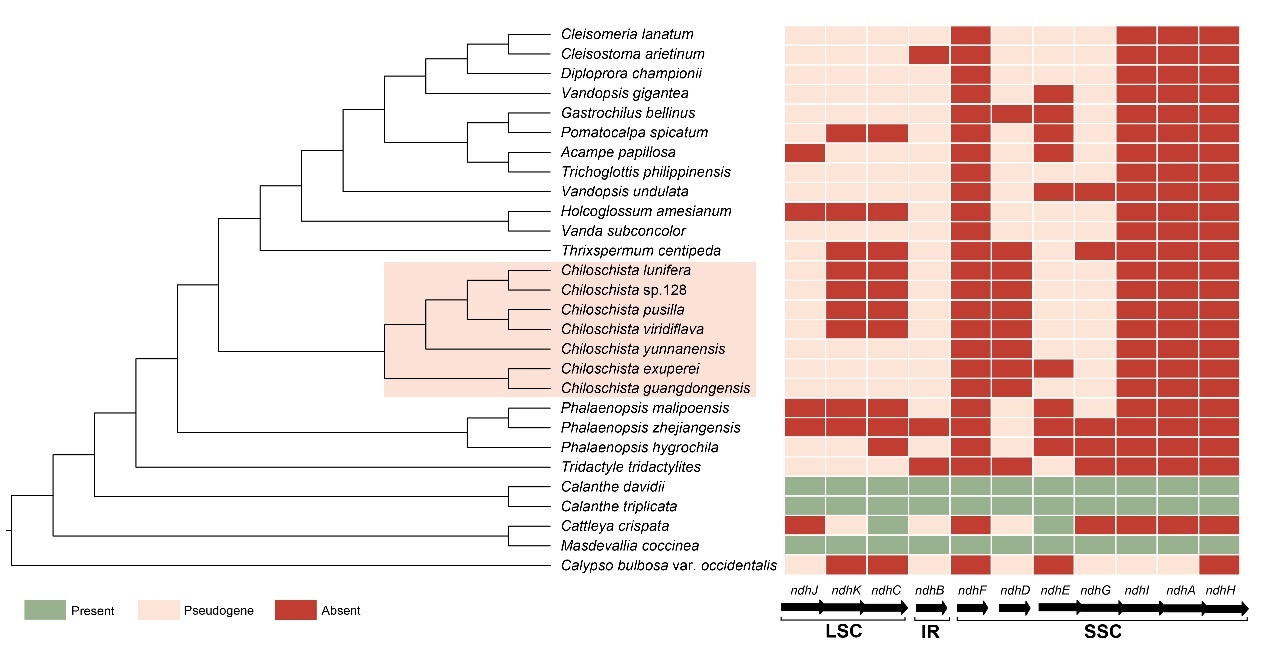
**

**Fig. S4.** The *ndh* genes loss across the subtribe Aeridinae.

**Table S1.** The statistics of raw data and plastome assembly results.

| Species | Size (Gb) | Number of reads | Aligned reads to plastome | *k*mer-coverage |
| --- | --- | --- | --- | --- |
| *Chiloschista exuperei* | 4.57 | 68,499,842 | 269,602 | 120.6 |
| *C. guangdongensis* | 12.20 | 178,491,193 | 295,927 | 111.2 |
| *C. lunifera* | 8.40 | 74,141,701 | 268,564 | 120.0 |
| *C. pusilla* | 9.22 | 80,738,477 | 284,501 | 111.9 |
| *C.* sp. 128 | 8.22 | 72,089,735 | 284,997 | 122.8 |
| *C. viridiflava* | 7.65 | 72,260,947 | 280,853 | 108.5 |
| *C. yunnanensis* | 4.35 | 82,523,887 | 267,364 | 121.8 |

**Table S2.** The description small inversion sequences of seven *Chiloschista*.

| **SI #** | **Type** | **Species** | **Base** | **Loop** | **Stem length** | **Loop length** | **dG** |
| --- | --- | --- | --- | --- | --- | --- | --- |
| 1 | A | *Chiloschista exuperei* | ATATGATGATATATACAATATATCATCATAT | ACA | 14 | 3 | -9.07 |
| 1 | A | *C. lunifera* | ATATGATGATATATACAATATATCATCATAT | ACA | 14 | 3 | -9.07 |
| 1 | A | *C. pusilla* | ATATGATGATATATACAATATATCATCATAT | ACA | 14 | 3 | -9.07 |
| 1 | A | *C.* sp. 128 | ATATGATGATATATACAATATATCATCATAT | ACA | 14 | 3 | -9.07 |
| 1 | A | *C. viridiflava* | ATATGATGATATATACAATATATCATCATAT | ACA | 14 | 3 | -9.07 |
| 1 | A | *C. yunnanensis* | ATATGATGATATATTGTATATATCATCATAT | ACA | 14 | 3 | -9.07 |
| 2 | A | *C. exuperei* | AGATTTTATTCTGAAAATCA | ATTCTG | 7 | 6 | -3.08 |
| 2 | A | *C. guangdongensis* | AGATTTTATTCTGAAAATCA | ATTCTG | 7 | 6 | -3.08 |
| 3 | A | *C. exuperei* | TAAAGAAGAAAGAATTCTATTCTTTCTTCTTTA | TCT | 15 | 3 | -12.36 |
| 3 | A | *C. lunifera* | TAAAGAAGAAAGAATTCTATTCTTTCTTCTTTA | TCT | 15 | 3 | -12.36 |
| 3 | A | *C. pusilla* | TAAAGAAGAAAGAATTCTATTCTTTCTTCTTTA | TCT | 15 | 3 | -12.36 |
| 3 | A | *C. viridiflava* | TAAAGAAGAAAGAATTCTATTCTTTCTTCTTTA | TCT | 15 | 3 | -12.36 |
| 3 | B | *C. yunnanensis* | TAAAGAAGAAAGAATAGAATTCTTTCTTCTTTA | AGA | 15 | 3 | -12.36 |
| 4 | A | *C. exuperei* | TCTTTTCACTTTTCTATAGATTTGATAGATCTATAGAAAAGTGAAAAGA | TTGATAG | 21 | 7 | -19.19 |
| 4 | A | *C. guangdongensis* | TCTTTTCACTTTTCTATAGATTTGATAGATCTATAGAAAAGTGAAAAGA | TTGATAG | 21 | 7 | -19.19 |
| 4 | A | *C. lunifera* | TCTTTTCACTTTTCTATAGATTTGATAGATCTATAGAAAAGTGAAAAGA | TTGATAG | 21 | 7 | -19.19 |
| 4 | A | *C.* sp. 128 | TCTTTTCACTTTTCTATAGATTTGATAGATCTATAGAAAAGTGAAAAGA | TTGATAG | 21 | 7 | -19.19 |
| 4 | A | *C. yunnanensis* | TCTTTTCACTTTTCTATAGATTTGATAGATCTATAGAAAAGTGAAAAGA | TTGATAG | 21 | 7 | -19.19 |
| 5 | A | *C. exuperei* | TGGCTCGGCTATCTCGCCTAGCCGAGCCA | TCTCGCC | 11 | 7 | -12.17 |
| 5 | A | *C. guangdongensis* | TGGCTCGGCTATCTCGCCTAGCCGAGCCA | TCTCGCC | 11 | 7 | -12.17 |
| 5 | A | *C. lunifera* | TGGCTCGGCTATCTCGCCTAGCCGAGCCA | TCTCGCC | 11 | 7 | -12.17 |
| 5 | A | *C. pusilla* | TGGCTCGGCTATCTCGCCTAGCCGAGCCA | TCTCGCC | 11 | 7 | -12.17 |
| 5 | A | *C.* sp. 128 | TGGCTCGGCTATCTCGCCTAGCCGAGCCA | TCTCGCC | 11 | 7 | -12.17 |
| 5 | A | *C. viridiflava* | TGGCTCGGCTATCTCGCCTAGCCGAGCCA | TCTCGCC | 11 | 7 | -12.17 |
| 5 | A | *C. yunnanensis* | TGGCTCGGCTATCTCGCCTAGCCGAGCCA | TCTCGCC | 11 | 7 | -12.17 |
| 6 | A | *C. exuperei* | GGCCCAATCTTTCTTTTTTTGAGGAAAAGATTGGGCC | CTTTTTTTGAGGA | 12 | 13 | -11.57 |
| 6 | B | *C. guangdongensis* | GGCCCAATCTTTTCCTCAAAAAAAGAAAGATTGGGCC | TCCTCAAAAAAAG | 12 | 13 | -11.67 |
| 6 | B | *C. lunifera* | GGCCCAATCTTTTCCTCAAAAAAAGAAAGATTGGGCC | TCCTCAAAAAAAG | 12 | 13 | -11.67 |
| 6 | A | *C. pusilla* | GGCCCAATCTTTCTTTTTTTGAGGAAAAGATTGGGCC | CTTTTTTTGAGGA | 12 | 13 | -11.57 |
| 6 | A | *C.* sp. 128 | GGCCCAATCTTTCTTTTTTTGAGGAAAAGATTGGGCC | CTTTTTTTGAGGA | 12 | 13 | -11.57 |
| 6 | A | *C. viridiflava* | GGCCCAATCTTTCTTTTTTTGAGGAAAAGATTGGGCC | CTTTTTTTGAGGA | 12 | 13 | -11.57 |
| 6 | A | *C. yunnanensis* | GGCCCAATCTTTCTTTTTTTGAGGAAAAGATTGGGCC | CTTTTTTTGAGGA | 12 | 13 | -11.57 |
| 7 | A | *C. exuperei* | TTTCTATCTTTACCTTTCAAAACACCTTTTTTGTTTTGAAAGGTAAAGATAGAAA | CCTTTTT | 24 | 7 | -22.35 |
| 7 | A | *C. guangdongensis* | TTTCTATCTTTACCTTTCAAAACACCTTTTTTGTTTTGAAAGGTAAAGATAGAAA | CCTTTTT | 24 | 7 | -22.35 |
| 7 | A | *C. lunifera* | TTTCTATCTTTACCTTTCAAAACACCTTTTTTGTTTTGAAAGGTAAAGATAGAAA | CCTTTTT | 24 | 7 | -22.35 |
| 7 | B | *C. pusilla* | TTTCTATCTTTACCTTTCAAAACAAAAAAGGTGTTTTGAAAGGTAAAGATAGAAA | AAAAAGG | 24 | 7 | -22.55 |
| 7 | B | *C.* sp. 128 | TTTCTATCTTTACCTTTCAAAACAAAAAAGGTGTTTTGAAAGGTAAAGATAGAAA | AAAAAGG | 24 | 7 | -22.55 |
| 7 | B | *C. viridiflava* | TTTCTATCTTTACCTTTCAAAACAAAAAAGGTGTTTTGAAAGGTAAAGATAGAAA | AAAAAGG | 24 | 7 | -22.55 |
| 7 | A | *C. yunnanensis* | TTTCTATCTTTACCTTTCAAAACACCTTTTTTGTTTTGAAAGGTAAAGATAGAAA | CCTTTTT | 24 | 7 | -22.35 |
| 8 | A | *C. guangdongensis* | GATACAAGACGACACAAGAAAAGGATTTTCTTGTGTCGTCTTGTATC | GGA | 22 | 3 | -23.95 |
| 8 | A | *C. lunifera* | GATACAAGACGACACAAGAAAAGGATTTTCTTGTGTCGTCTTGTATC | GGA | 22 | 3 | -23.95 |
| 8 | A | *C. pusilla* | GATACAAGACGACACAAGAAAAGGATTTTCTTGTGTCGTCTTGTATC | GGA | 22 | 3 | -23.95 |
| 8 | A | *C. viridiflava* | GATACAAGACGACACAAGAAAAGGATTTTCTTGTGTCGTCTTGTATC | GGA | 22 | 3 | -23.95 |
| 8 | A | *C. yunnanensis* | GATACAAGACGACACAAGAAAATCCTTTTCTTGTGTCGTCTTGTATC | GGA | 22 | 3 | -23.95 |
| 9 | A | *C. exuperei* | AAGAGTTAAGAACTCAGCGGGGCCTTACCCCGCTGAGTTCTTACTCTT | CCTTA | 22 | 5 | -21.79 |
| 9 | A | *C. guangdongensis* | AAGAGTTAAGAACTCAGCGGGGCCTTACCCCGCTGAGTTCTTACTCTT | CCTTA | 22 | 5 | -21.79 |
| 9 | B | *C. lunifera* | AAGAGTTAATAACTCAGCGGGGTAAGGCCCCGCTGAGTTATTACTCTT | TAAGG | 22 | 5 | -20.57 |
| 9 | B | *C. pusilla* | AAGAGTTAATAACTCAGCGGGGTAAGGCCCCGCTGAGTTATTACTCTT | TAAGG | 22 | 5 | -20.57 |
| 9 | B | *C.* sp. 128 | AAGAGTTAATAACTCAGCGGGGTAAGGCCCCGCTGAGTTATTACTCTT | TAAGG | 22 | 5 | -20.57 |
| 9 | B | *C. viridiflava* | AAGAGTTAATAACTCAGCGGGGTAAGGCCCCGCTGAGTTATTACTCTT | TAAGG | 22 | 5 | -20.57 |
| 9 | A | *C. yunnanensis* | AAGAGTTAAGAACTCAGCGGGGCCTTACCCCGCTGAGTTCTTACTCTT | CCTTA | 22 | 5 | -21.79 |
| 10 | A | *C. exuperei* | AAAAAAAATCAAATGAAAATATTCAATATTTTCATTTGATTTTTTTT | TCA | 21 | 3 | -18.15 |
| 10 | A' | *C. guangdongensis* | GAAAAAAATCAAATGAAAATATTCAATATTTTCATTTGATTTTTTTT | TCA | 21 | 3 | -17.44 |
| 10 | A | *C. lunifera* | AAAAAAAATCAAATGAAAATATTGAATATTTTCATTTGATTTTTTTT | TCA | 21 | 3 | -18.15 |
| 10 | A | *C. pusilla* | AAAAAAAATCAAATGAAAATATTGAATATTTTCATTTGATTTTTTTT | TCA | 21 | 3 | -18.15 |
| 10 | A | *C.* sp. 128 | AAAAAAAATCAAATGAAAATATTGAATATTTTCATTTGATTTTTTTT | TCA | 21 | 3 | -18.15 |
| 10 | A | *C. viridiflava* | AAAAAAAATCAAATGAAAATATTGAATATTTTCATTTGATTTTTTTT | TCA | 21 | 3 | -18.15 |
| 10 | A' | *C. yunnanensis* | GAAAAAAATCAAATGAAAATATTGAATATTTTCATTTGATTTTTTTT | TCA | 21 | 3 | -17.44 |
| 11 | A | *C. exuperei* | TTGAAGTAATGAGCCCCAATATGAATATCGGGGCTCATTACTTCAA | TGAA | 21 | 4 | -18.33 |
| 11 | A | *C. guangdongensis* | TTGAAGTAATGAGCCCCAATATGAATATCGGGGCTCATTACTTCAA | TGAA | 21 | 4 | -18.33 |
| 11 | B | *C. lunifera* | TTGAAGTAATGAGCCCCGATATTCATATTGGGGCTCATTACTTCAA | TTCA | 21 | 4 | -20.54 |
| 11 | B | *C. pusilla* | TTGAAGTAATGAGCCCCGATATTCATATTGGGGCTCATTACTTCAA | TTCA | 21 | 4 | -20.54 |
| 11 | B | *C.* sp. 128 | TTGAAGTAATGAGCCCCGATATTCATATTGGGGCTCATTACTTCAA | TTCA | 21 | 4 | -20.54 |
| 11 | B | *C. viridiflava* | TTGAAGTAATGAGCCCCGATATTCATATTGGGGCTCATTACTTCAA | TTCA | 21 | 4 | -20.54 |
| 11 | A | *C. yunnanensis* | TTGAAGTAATGAGCCCCAATATGAATATCGGGGCTCATTACTTCAA | TGAA | 21 | 4 | -18.33 |
| 12 | A | *C. exuperei* | TATCTAAGGAAGATCCAGAAGGGGATCTTCCTTAGATA | AGAAGG | 16 | 6 | -15.1 |
| 12 | B | *C. guangdongensis* | TATCTAAGGAAGATCCCCTTCTGGATCTTCCTTAGATA | CCTTCT | 16 | 6 | -14.9 |
| 12 | B | *C. lunifera* | TATCTAAGGAAGATCCCCTTCTGGATCTTCCTTAGATA | CCTTCT | 16 | 6 | -14.9 |
| 12 | B | *C. pusilla* | TATCTAAGGAAGATCCCCTTCTGGATCTTCCTTAGATA | CCTTCT | 16 | 6 | -14.9 |
| 12 | B | *C.* sp. 128 | TATCTAAGGAAGATCCCCTTCTGGATCTTCCTTAGATA | CCTTCT | 16 | 6 | -14.9 |
| 12 | B | *C. viridiflava* | TATCTAAGGAAGATCCCCTTCTGGATCTTCCTTAGATA | CCTTCT | 16 | 6 | -14.9 |
| 12 | B | *C. yunnanensis* | TATCTAAGGAAGATCCCCTTCTGGATCTTCCTTAGATA | CCTTCT | 16 | 6 | -14.9 |
| 13 | A | *C. exuperei* | TTTTGTACGTCCCCATGTCCCTCCCGTGTGGCGACATGGGGACGTACAAAAGG | TCCCGTGT | 21 | 8 | -21.1 |
| 13 | A' | *C. guangdongensis* | CTTTTGTACGTCCCCATGTCCCTCCCGTGTGGCGACATGGGGACGTACAAAAG | TCCCGTGT | 22 | 8 | -22.42 |
| 13 | A | *C. lunifera* | TTTTGTACGTCCCCATGTCCCTCCCGTGTGGCGACATGGGGACGTACAAAAGG | TCCCGTGT | 21 | 8 | -21.1 |
| 13 | A | *C. pusilla* | TTTTGTACGTCCCCATGTCCCTCCCGTGTGGCGACATGGGGACGTACAAAAGG | TCCCGTGT | 21 | 8 | -21.1 |
| 13 | A | *C.* sp. 128 | TTTTGTACGTCCCCATGTCCCTCCCGTGTGGCGACATGGGGACGTACAAAAGG | TCCCGTGT | 21 | 8 | -21.1 |
| 13 | A | *C. viridiflava* | TTTTGTACGTCCCCATGTCCCTCCCGTGTGGCGACATGGGGACGTACAAAAGG | TCCCGTGT | 21 | 8 | -21.1 |
| 13 | A | *C. yunnanensis* | TTTTGTACGTCCCCATGTCCCTCCCGTGTGGCGACATGGGGACGTACAAAAGG | TCCCGTGT | 21 | 8 | -21.1 |
| 14 | A | *C. exuperei* | AATTGTGCTTTTCAAAATAGAAAAGCACAATT | AAAATA | 20 | 6 | -11.99 |
| 14 | A | *C. guangdongensis* | AATTGTGCTTTTCAAAATAGAAAAGCACAATT | AAAATA | 20 | 6 | -11.99 |
| 14 | A | *C. lunifera* | AATTGTGCTTTTCAAAATAGAAAAGCACAATT | AAAATA | 20 | 6 | -11.99 |
| 14 | A | *C. pusilla* | AATTGTGCTTTTCAAAATAGAAAAGCACAATT | AAAATA | 20 | 6 | -11.99 |
| 14 | A | *C.* sp. 128 | AATTGTGCTTTTCAAAATAGAAAAGCACAATT | AAAATA | 20 | 6 | -11.99 |
| 14 | A | *C. viridiflava* | AATTGTGCTTTTCAAAATAGAAAAGCACAATT | AAAATA | 20 | 6 | -11.99 |
| 14 | A | *C. yunnanensis* | AATTGTGCTTTTCAAAATAGAAAAGCACAATT | AAAATA | 20 | 6 | -11.99 |

**Table S3.** The details information of long repeats.

|  | species | *C. exuperei* | *C. guangdongensis* | *C. lunifera* | *C. pusilla* | *C.* sp. 128 | *C. viridiflava* | *C. yunnanensis* |
| --- | --- | --- | --- | --- | --- | --- | --- | --- |
| 40+ | C | 0 | 0 | 0 | 0 | 0 | 0 | 0 |
|  | F | 1 | 1 | 0 | 0 | 0 | 0 | 0 |
|  | P | 4 | 4 | 5 | 6 | 3 | 6 | 5 |
|  | R | 0 | 0 | 0 | 0 | 0 | 0 | 0 |
| 30-39 | C | 0 | 0 | 0 | 0 | 0 | 0 | 0 |
|  | F | 2 | 1 | 2 | 1 | 1 | 1 | 1 |
|  | P | 6 | 5 | 6 | 5 | 6 | 5 | 5 |
|  | R | 1 | 1 | 0 | 1 | 1 | 1 | 1 |
| 20-29 | C | 0 | 1 | 1 | 2 | 1 | 2 | 1 |
|  | F | 19 | 17 | 18 | 18 | 17 | 17 | 15 |
|  | P | 25 | 25 | 22 | 20 | 19 | 20 | 22 |
|  | R | 7 | 14 | 8 | 9 | 5 | 9 | 10 |

Note: The “C, F, P, R” represented “Complement, Forward, Palindromic, Reverse”.

**Table S4.** The details information Small simple repeats. (Please see separate file)

**Table S5.** Comparison of non-synonymous (dN) / synonymous (dS) substitution rates among *Chiloschista* plastid protein-coding genes. (Please see separate file)

**Table S6.** The nucleotide diversity of seven *Chiloschista* plastome. (Please see separate file)

**Table S7.** The nucleotide diversity of 68 protein coding genes in *Chiloschista*. (Please see separate file)

**Table S8.** Source and voucher information for this study. Voucher specimens were deposited in the herbariums of Forestry College of Fujian Agriculture and Forestry University (FJFC).

| Species | Source | Voucher | GenBank accession numbers |
| --- | --- | --- | --- |
| *Chiloschista yunnanensis* | This study | MHLi or082 | OP953689 |
| *C. exuperei* | This study | MHLi or085 | OP953683 |
| *C. guangdongensis* | This study | MHLi or111 | OP953684 |
| *C. lunifera* | This study | MHLi or120 | OP953685 |
| *C. pusilla* | This study | MHLi or112 | OP953686 |
| *C.* sp. 128 | This study | MHLi or113 | OP953687 |
| *C. viridiflava* | This study | MHLi or082 | OP953688 |
| *Acampe papillosa* | Liu et al., 2020 | Liu3246 | MN124418 |
| *Cleisomeria lanatum* | Liu et al., 2020 | Lior001 | MN124442 |
| *Cleisostoma arietinum* | Liu et al., 2020 | Liu6991 | MN124420 |
| *Diploprora championii* | Liu et al., 2020 | Liu4480 | MN124409 |
| *Gastrochilus bellinus* | Liu et al., 2020 | Lior011 | MN124427 |
| *Holcoglossum amesianum* | Liu et al., 2020 | 9419 | MK442924 |
| *Phalaenopsis hygrochila* | Liu et al., 2020 | Liu3336 | MN124430 |
| *Phalaenopsis malipoensis* | Hu et al., 2022 | - | OL623704 |
| *Phalaenopsis zhejiangensis* | Jiang et al., 2021 | CHS2020029 | MZ326749 |
| *Pomatocalpa spicatum* | Liu et al., 2020 | Liu4589 | MN124411 |
| *Thrixspermum centipeda* | Liu et al., 2020 | MH Li081 | MW057769 |
| *Trichoglottis philippinensis* | Liu et al., 2020 | Lior075 | MN124404 |
| *Vanda subconcolor* | Liu et al., 2020 | MH Li or086 | MT180955 |
| *Vandopsis gigantea* | Liu et al., 2020 | Liu7990 | MN124403 |
| *Vandopsis undulata* | Liu et al., 2020 | Lior078 | MN124402 |
| **Outgroup** |  |  |  |
| *Calanthe davidii* | Dong et al., 2018 | - | MG925365 |
| *Calanthe triplicata* | Yang et al., 2014 | - | KF753635 |
| *Calypso bulbosa* var. *occidentalis* | Barrett et al., 2018 | CFB 349 OR | MG874037 |
| *Cattleya crispata* | da Rocha et al., 2016 | - | KP168671 |
| *Masdevallia coccinea* | Kim et al., 2015 | - | KP205432 |
| *Tridactyle tridactylites* | D'haijère et al., 2022 | - | MW760855 |

Barrett CF, Wicke S, Sass C (2018) Dense infraspecific sampling reveals rapid and independent trajectories of plastome degradation in a heterotrophic orchid complex. New Phytol 218:1192–1204. https://doi.org/10.1111/nph.15072

D’haijère T, Kaymak E, Boom AF, et al (2022) Diversification of the orchid genus *Tridactyle*: Origin of endemism on the oceanic islands of São Tomé & Príncipe in the Gulf of Guinea. J Biogeogr 49:523–536. https://doi.org/10.1111/jbi.14324

Dong WL, Wang RN, Zhang NY, et al (2018) Molecular evolution of chloroplast genomes of orchid species: Insights into phylogenetic relationship and adaptive evolution. Int J Mol Sci 19:. https://doi.org/10.3390/ijms19030716

Hu M, Tong E, Zhang Y, et al (2022) The complete chloroplast sequence of *Phalaenopsis* *malipoensis*, a rare orchidaceae species in China. Mitochondrial DNA Part B Resour 7:1489–1491. https://doi.org/10.1080/23802359.2022.2107453

Jiang M, Zhu Y, Wu Q, Zhang H (2021) Complete chloroplast genome of a rare and endangered plant species *Phalaenopsis zhejiangensis*: genomic features and phylogenetic relationship within Orchidaceae. Mitochondrial DNA Part B Resour 6:2872–2879. https://doi.org/10.1080/23802359.2021.1972049

Kim HT, Kim JS, Moore MJ, et al (2015) Seven new complete plastome sequences reveal rampant independent loss of the ndh gene family across orchids and associated instability of the inverted repeat/small single-copy region boundaries. PLoS One 10:. https://doi.org/10.1371/journal.pone.0142215

Liu D, Tu X, Zhao Z, et al (2020) Plastid phylogenomic data yield new and robust insights into the phylogeny of *Cleisostoma–Gastrochilus* clades (Orchidaceae, Aeridinae). Mol Phylogenet Evol 145:106729. https://doi.org/10.1016/j.ympev.2019.106729

Yang J-B, Li D-Z, Li H-T (2014) Highly effective sequencing whole chloroplast genomes of angiosperms by nine novel universal primer pairs. Mol Ecol Resour 14:n/a-n/a. https://doi.org/10.1111/1755-0998.12251
